# The Essential Oil Compositions of *Ambrosia acanthicarpa* Hook., *Artemisia ludoviciana* Nutt., and *Gutierrezia sarothrae* (Pursh) Britton & Rusby (Asteraceae) from the Owyhee Mountains of Idaho

**DOI:** 10.3390/molecules29061383

**Published:** 2024-03-20

**Authors:** Kathy Swor, Ambika Poudel, Prabodh Satyal, William N. Setzer

**Affiliations:** 1 Independent Researcher, 1432 W. Heartland Dr., Kuna, ID 83634, USA; 2 Aromatic Plant Research Center, 230 N 1200 E, Suite 100, Lehi, UT 84043, USApsatyal@aromaticplant.org (P.S.); 3 Department of Chemistry, University of Alabama in Huntsville, Huntsville, AL 35899, USA

**Keywords:** ragweed, burweed, bur-sage, white sage, silver wormwood, broom snakeweed, gas chromatography, enantiomers

## Abstract

As part of our interest in the volatile phytoconstituents of aromatic plants of the Great Basin, we have obtained essential oils of *Ambrosia acanthicarpa* (three samples), *Artemisia ludoviciana* (12 samples), and *Gutierrezia sarothrae* (six samples) from the Owyhee Mountains of southwestern Idaho. Gas chromatographic analyses (GC-MS, GC-FID, and chiral GC-MS) were carried out on each essential oil sample. The essential oils of *A. acanthicarpa* were dominated by monoterpene hydrocarbons, including α-pinene (36.7–45.1%), myrcene (21.6–25.5%), and β-phellandrene (4.9–7.0%). Monoterpene hydrocarbons also dominated the essential oils of *G. sarothrae*, with β-pinene (0.5–18.4%), α-phellandrene (2.2–11.8%), limonene (1.4–25.4%), and (*Z*)-β-ocimene (18.8–39.4%) as major components. The essential oils of *A. ludoviciana* showed wide variation in composition, but the relatively abundant compounds were camphor (0.1–61.9%, average 14.1%), 1,8-cineole (0.1–50.8%, average 11.1%), (*E*)-nerolidol (0.0–41.0%, average 6.8%), and artemisia ketone (0.0–46.1%, average 5.1%). This is the first report on the essential oil composition of *A. acanthicarpa* and the first report on the enantiomeric distribution in an *Ambrosia* species. The essential oil compositions of *A. ludoviciana* and *G. sarothrae* showed wide variation in composition in this study and compared with previous studies, likely due to subspecies variation.

## 1. Introduction

**Figure 1:**
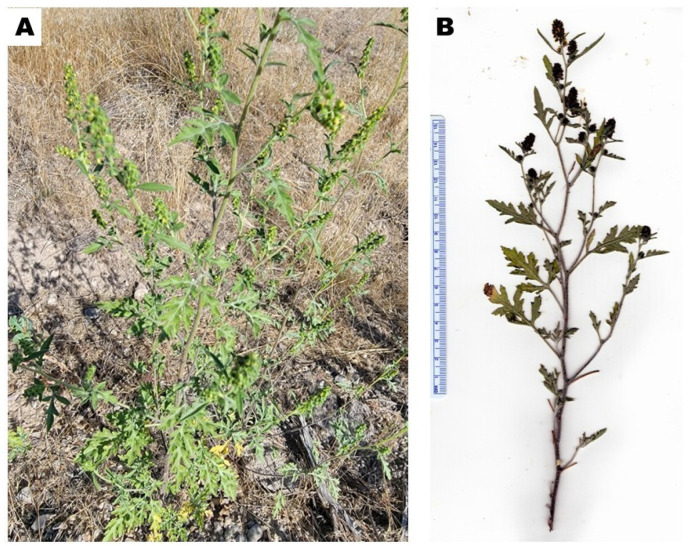
*Ambrosia acanthicarpa*. (**A**): Photograph of *A. acanthicarpa* (K. Swor). (**B**): Scan of pressed plant (W.N. Setzer).

*Ambrosia acanthicarpa* Hook. (bur ragweed, burweed, bur-sage) is an annual member of the Asteraceae. The leaves are deltoid to narrowly lanceolate, to 8 cm long and 6 cm wide, pinnately to tripinnately lobed, and both leaf surfaces are green and have a dense covering of short, matted hairs [1]. The stems are grayish-green, with stiff, bristly hairs (Figure 1). In the United States, the plant ranges from eastern Washington and Oregon, eastern and southern California, east to western North Dakota, South Dakota, Nebraska, and Kansas, and the panhandle of Texas [1].

Previous phytochemical investigations of *A. acanthicarpa* have revealed the plant to be a source of sesquiterpene lactones, including artemisiifolin, chamissonin, psilostachyin C, confertiflorin, deacetylconfertiflorin, and cumambrin B [2,3]. As far as we are aware, there have been no previous studies on the essential oil of this plant.

**Figure 2:**
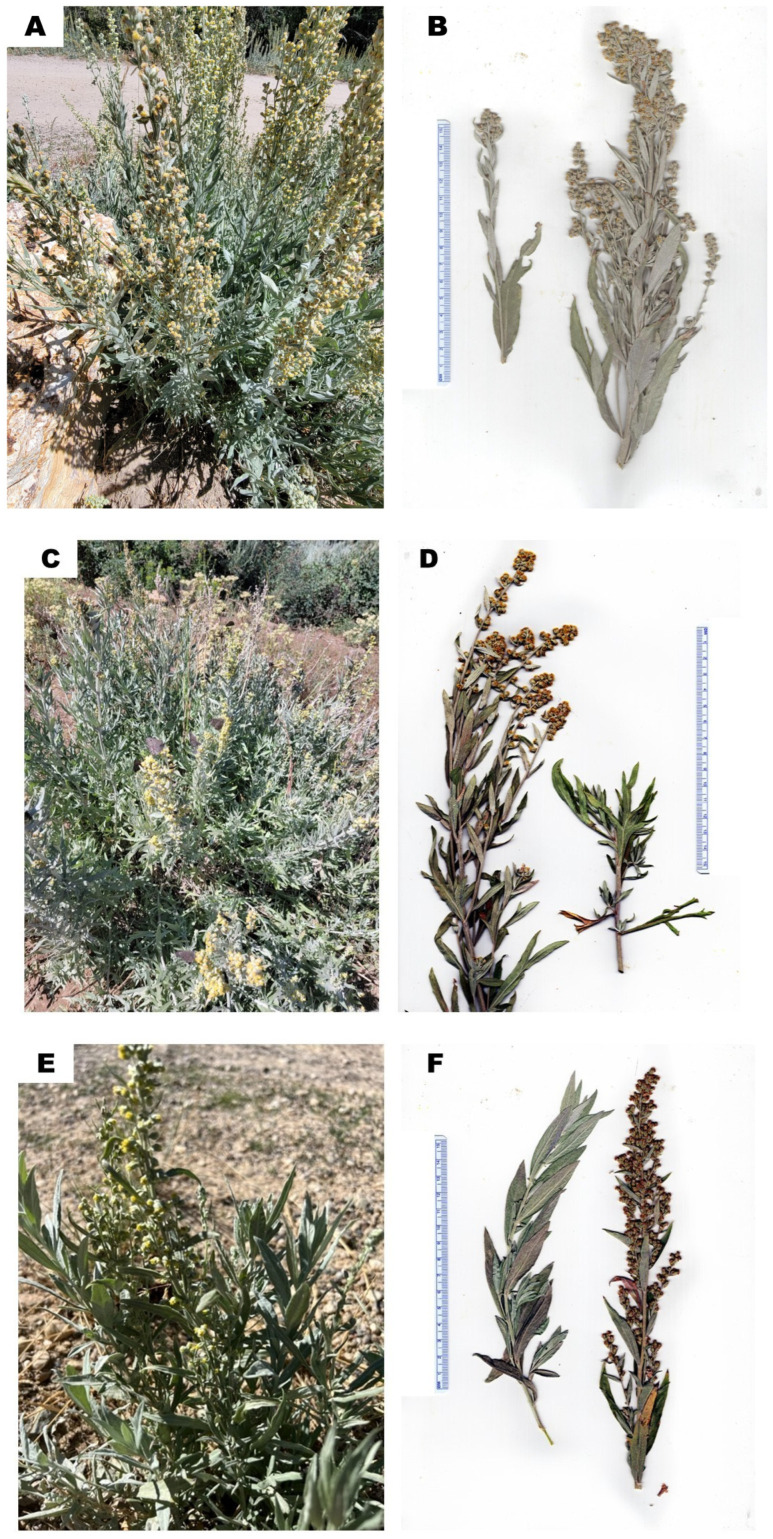

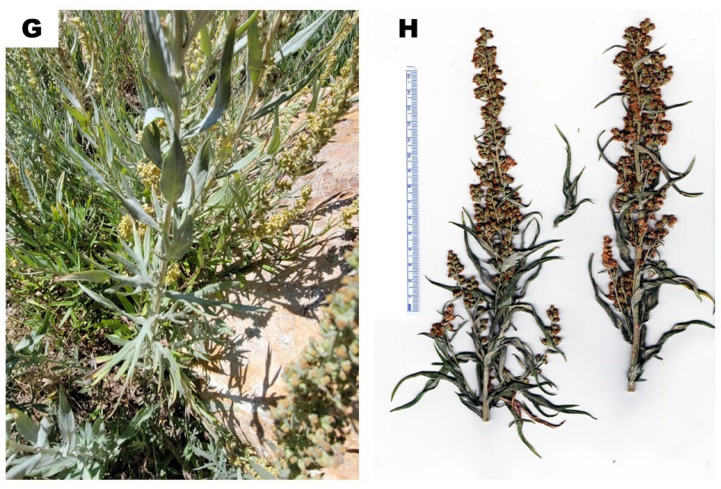
*Artemisia ludoviciana*. (**A**): Photograph of *A. ludoviciana*, plant B1 (K. Swor). (**B**): Scan of pressed plant B1 (W.N. Setzer). (**C**): Photograph of *A. ludoviciana*, plant C1 (K. Swor). (**D**): Scan of pressed plant C1 (W.N. Setzer). (**E**): Photograph of *A. ludoviciana*, plant T1 (K. Swor). (**F**): Scan of pressed plant T1 (W.N. Setzer). (**G**): Photograph of *A. ludoviciana*, plant U1 (K. Swor). (**H**): Scan of pressed plant U1 (W.N. Setzer).

*Artemisia ludoviciana* Nutt. (white sage, silver wormwood, Asteraceae) is a perennial herb, 30–70 cm tall, with a sagebrush odor. The leaves are alternate, entire, or lobed, 3–11 cm long, and up to 1.5 cm wide, with a dense covering of short, matted hairs. The plant flowers from August through September, producing numerous nodding flower heads (Figure 2) [4,5]. The plant is highly polymorphic and there are several subordinate taxa. World Flora Online currently lists seven subspecies, including *Artemisia ludoviciana* subsp. *albula* (Wooton) D.D. Keck, *Artemisia ludoviciana* subsp. *candicans* (Rydb.) D.D. Keck, *Artemisia ludoviciana* subsp. *incompta* (Nutt.) D.D. Keck, *Artemisia ludoviciana* subsp. *lindleyana* (Besser) Lesica, *Artemisia ludoviciana* subsp. *ludoviciana*, *Artemisia ludoviciana* subsp. *mexicana* (Spreng.) D.D. Keck, and *Artemisia ludoviciana* subsp. *redolens* (A. Gray) D.D. Keck. [6]. Of these, *A. ludoviciana* subsp. *ludoviciana* [7], *A. ludoviciana* subsp. *candicans* [8], and *A. ludoviciana* subsp. *incompta* [9] are known to occur in Idaho. However, these subspecies are variable morphologically, intergrade between taxa, and recognition of the discreet taxa is therefore difficult and questionable. *Artemisia ludoviciana* is widespread throughout western North America, ranging from Ontario and Michigan, west to British Columbia, and south through Texas, Louisiana, California, and Mexico [4,5].

The plant is used in traditional herbal medicine throughout its range. In Mexico, the people use an infusion of the aerial parts of *A. ludoviciana* to treat diarrhea, parasitic diseases, painful conditions, and diabetes [10,11]. In the Great Basin of North America, the Paiute people used a decoction of *A. ludoviciana* as a bath for aching feet, as a poultice for rheumatism or other aches, to treat rashes and skin eruptions, and to relieve diarrhea, while the Shoshone took the plant for coughs, colds, and influenza, and to stop diarrhea [12].

*Artemisia ludoviciana* has proven to be a rich source of sesquiterpene lactones, including ludovicin-A, -B, -C, and -D [13], ludalbin [14], anthemidin [15], arteludovicinolide-A, -B, -C, and -D [16], douglanin, santamarin, arglanine, artemorin, chrysartemin B, armefolin, and ridentin [17]; flavonoids, including jaceosidin, tricin, hispidulin, chrysoeriol, apigenin, axillarin, eupafolin, selagin, luteolin [18], eupatilin, and jaceosidin [17]; and the spiroketals 2-(2-thienylidene)-1,6-dioxaspiro[4.5]dec-3-ene and 5-[[5-(1,6-dioxaspiro[4.5]dec-3-en-2-ylidenemethyl)-2-thienyl]-2-thienylmethyl]-2-furanbutanol [19]. There have been previous investigations on the essential oil composition of *A. ludoviciana* from different geographical locations, including Utah, USA [20], Huixquilucan, State of Mexico, Mexico [21], Alberta, Canada [22], Ozumba, State of Mexico, Mexico [23], South Dakota, USA [24], Istanbul, Türkiye (cultivated) [25], and Wyoming, USA [26].

**Figure 3:**
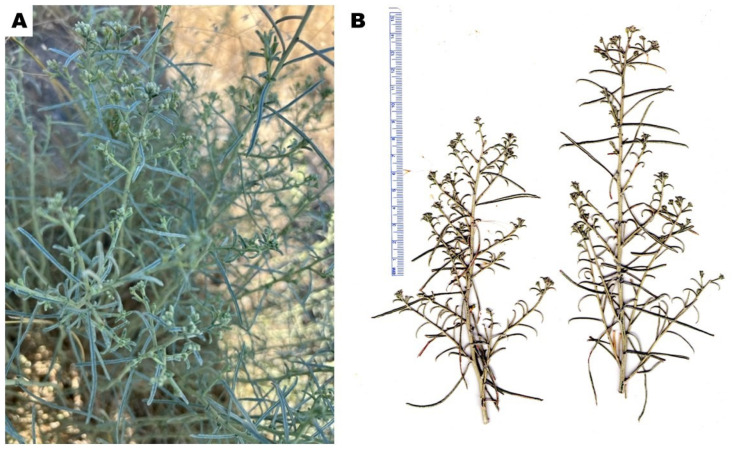
*Gutierrezia sarothrae*. (**A**): Photograph of *G. sarothrae* (K. Swor). (**B**): Scan of pressed plant (W.N. Setzer).

*Gutierrezia sarothrae* (Pursh) Britton & Rusby (syn. *Xanthocephalum sarothrae* (Pursh) Shinners, broom snakeweed, Asteraceae) is an herbaceous shrub, 10–60 cm in height; stems are green to brown; leaves are alternate, lanceolate, sometimes filiform, green, up to 4–55 mm long, and 0.3–5 mm wide (Figure 3). The plant flowers July-November, producing numerous small, bright-yellow flowers [27,28,29]. *Gutierrezia sarothrae* ranges throughout western North America, from eastern Oregon and Washington, eastern and southern California east to the Great Plains, and from southern Alberta, Saskatchewan, and Manitoba, south into northern Mexico [27,30].

Diterpenoids, including polyalthic acid, daniellic acid, nivenolide, and gutierrezial [31,32,33], and flavonoids, including apigenin, luteolin, calycopterin, jaceidin, sudachitin, and sarothrin [34], have been isolated and characterized from *G. sarothrae* extracts. The plant is an invasive weed and there have been several reports on the toxic effects of livestock grazing on *G. sarothrae*, causing abortions [30]. The abortifacient compounds in *G. sarothrae* are not known, but diterpene acids may be responsible [30]. There have been previous investigations of the essential oil composition of *G. sarothrae* from New Mexico and from Utah, USA [35,36,37].

As part of our ongoing efforts to obtain and characterize essential oils from the Asteraceae of the Great Basin [38], the purpose of this study is to obtain and chemically characterize the essential oils of *A. acanthicarpa*, *A. ludoviciana*, and *G. sarothrae* from southwestern Idaho. Although there have been previous investigations on the essential oils of *A. ludoviciana* and *G. sarothrae*, this present study is focused on the species from southwestern Idaho and also includes enantioselective gas chromatographic analyses to determine the enantiomeric distributions of chiral terpenoid constituents in these essential oils.

## 2. Results and Discussion

### 2.1. Ambrosia acanthicarpa

Hydrodistillation of the aerial parts of *A. acanthicarpa* yielded salmon-colored essential oils with a fish-like odor in yields of 4.36–5.01%. Gas chromatographic analysis led to identification of 135 components representing 97.6–98.0% of the total compositions (Table 1). Monoterpene hydrocarbons dominated the essential oils with α-pinene (36.7–45.1%), myrcene (21.6–25.5%), and β-phellandrene (4.9–7.0%) as the major components.

Although there have been no previous investigations of *A. acanthicarpa* essential oil compositions, there have been several reports on chemical compositions of other *Ambrosia* essential oils. Cicció and Chaverri have examined *Ambrosia cumanensis* Kunth from Costa Rica and have summarized the major components in *Ambrosia* essential oils published prior to 2015 [39]. A summary of the major components of *Ambrosia* essential oils published since 2015 is shown in Table 2. *Ambrosia* essential oils are typically dominated by sesquiterpene hydrocarbons and/or oxygenated monoterpenoids, in contrast to *A. acanthicarpa*, which was dominated by monoterpene hydrocarbons.

Enantioselective GC-MS was carried out on the three *A. acanthicarpa* samples (Table 3). The (−)-enantiomers were dominant for α-thujene (73.8–77.9%), α-pinene (99.3–99.4%), camphene (93.5–95.8%), β-pinene (86.5–87.9%), (*E*)-β-caryophyllene (100%), and germacrene D (93.5–100.0%). (+)-β-Phellandrene (97.0–98.2%), and (+)-δ-cadinene were the predominant enantiomers. Sabinene and limonene occurred in virtually racemic mixtures. Only one peak was observed for α-phellandrene (samples #2 and #3), but the RI is consistent with (+)-α-phellandrene. Likewise, only one peak was observed for lavandulol and the RI is consistent with (−)-lavandulol. One peak was observed for borneol, but the RI (1337) was in between (−)-borneol (1335) and (+)-borneol (1340), so the enantiomer cannot be assigned. Only one peak was observed for β-bisabolene, but the RI is consistent with the (+)-enantiomer. As far as we are aware, there have been no previous investigations on the enantiomeric distribution of essential oil components of *Ambrosia* species.

### 2.2. Artemisia ludoviciana

Essential oils from the aerial parts of *A. ludoviciana* were obtained from 12 individual plants in yields ranging from 0.580% to 3.306% (average yield 2.17%). Gas chromatographic analysis of the 12 *A. ludoviciana* essential oil samples (GC-FID, GC-MS) led to identification of 232 compounds accounting for 79.2–99.0% of the total compositions (Table 4). Although the essential oils were qualitatively similar, there were large quantitative differences in the compositions. The compounds found in relatively abundant concentrations were camphor (0.1–61.9%, average 14.1%), 1,8-cineole (0.1–50.8%, average 11.1%), (*E*)-nerolidol (0.0–41.0%, average 6.8%), artemisia ketone (0.0–46.1%, average 5.1%), linalool (0.1–19.9%, average 4.1%), and santolina triene (trace-18.8%, average 4.0%). There were also several unidentified components with relatively high concentrations. The mass spectra of the major unidentified compounds are available as supplementary material (Supplementary Figure S1).

**Figure 4:**
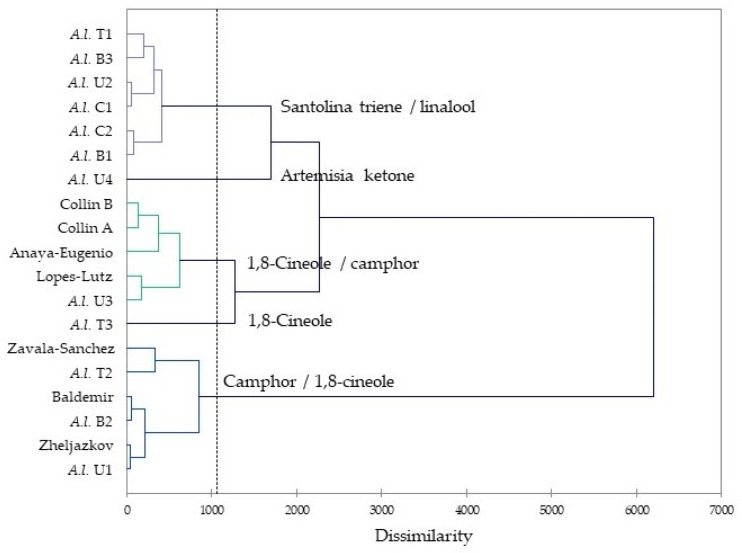
Dendrogram obtained by agglomerative hierarchical cluster (AHC) analysis of *Artemisia ludoviciana* essential oil compositions from this work and previously published investigations (Collin A, B [24], Anaya-Eugenio [23], Lopes-Lutz [22], Zavala-Sanchez [21], Baldemir [25], Zheljazkov [26]).

Previous investigations of *A. ludoviciana* essential oil showed camphor to be abundant (15.9–46.2%, average 30.0%), followed by 1,8-cineole (0.7–26.2%, average 15.2%), borneol (0.9–18.0%, average 8.5%), and α-terpineol (0.2–18.0%, average 3.3%) [21,22,23,24,25,26]. In order to place the volatile phytochemistry of this plant into perspective, an agglomerative hierarchical cluster (AHC) analysis was carried out using the major components in the essential oils from this work as well as the previously published compositions. The cluster analysis shows five possible groupings based on chemical compositions (Figure 4). The chemical groupings are (1) a santolina triene/linalool cluster, (2) a camphor/1,8-cineole cluster, (3) a 1,8-cineole “cluster” (one sample only), (4) a 1,8-cineole/camphor cluster, and (5) an artemisia ketone “cluster” (one sample only). Surprisingly, the 12 Idaho samples are distributed throughout the five clusters, demonstrating the phytochemical diversity of this plant species even within a small geographical range.

Enantioselective GC-MS analyses were carried out on the 12 *A. ludoviciana* essential oil samples (Table 5). Pure enantiomers (enantiomeric excess, ee = 100%) were found for (−)-α-thujene, (−)-lavandulol, (−)-borneol, (−)-α-copaene, (−)-(*E*)-β-caryophyllene, (−)-germacrene D, and (+)-δ-cadinene. The levorotatory enantiomers predominated for α-pinene (average ee = 46.0%), camphene (average ee = 94.4%), β-pinene (average ee = 73.6%), *cis*-sabinene hydrate (average ee = 70.2%), and *trans*-sabinene hydrate (average ee = 29.9%). Several monoterpenoid constituents did not show consistent enantiomeric distribution. Sabinene was mostly dominated by (−)-sabinene, but one sample (*A.l.* C1) had (+)-sabinene as the major enantiomer. Likewise, (−)-terpinen-4-ol dominated most essential oil samples, but sample *A.l.* T2 showed a slight excess of (+)-terpinen-4-ol. Similarly, (−)-α-terpineol predominated in most samples, but sample *A.l.* B2 showed an excess of (+)-α-terpineol. In the case of limonene, four samples had (−)-limonene predominating, while two samples had (+)-limonene as the major enantiomer. There was no consistency in the enantiomeric distribution of linalool. In the case of camphor, (−)-camphor predominated except for one sample (*A.l.* T3). Note, however, that camphor was abundant in samples *A.l.* C2, *A.l* T1, *A.l.* T2, and *A.l.* U2, so separation of the enantiomers was likely not possible. A similar situation existed for (*E*)-nerolidol; two samples (*A.l.* B1 and *A.l.* B3) had high concentrations of (*E*)-nerolidol, precluding enantiomeric separation.

There have been several reports that investigated the enantiomeric distributions of monoterpenoids in *Artemisia* essential oils. Consistent with the enantiomeric distributions for α-pinene, camphene, and β-pinene, the (−)-enantiomers predominated in the essential oil of *Artemisia annua* L. [46] and *Artemisia tridentata* subsp. *vaseyana* (Rydb.) Beetle [47]. Limonene enantiomers were variable in *A. ludoviciana* (this work), but (+)-limonene was dominant in *Artemisia arborescens* L. [48] and (−)-limonene was dominant in *A. annua* [46]. Linalool enantiomeric distribution was inconsistent in *A. ludoviciana* (this work), while (+)-linalool predominated in *A. arborescens* [48,49]. (+)-Terpinen-4-ol and (−)-α-terpineol were the dominant enantiomers in *A. arborescens* [48,49]. Interestingly, (−)-terpinen-4-ol was the dominant enantiomer in *Artemisia tridentata* Nutt. subsp. *tridentata* and *A. tridentata* subsp. *vaseyana*, but (+)-terpinen-4-ol dominated the essential oil of *Artemisia tridentata* subsp. *wyomingensis* Beetle & A.L. Young [47]. However, (−)-α-terpineol was the dominant enantiomer in *A. tridentata* subsp. *vaseyana* [47].

Consistent with the observations in *A. ludoviciana*, (−)-camphor was the dominant enantiomer in *A. arborescens* from Algeria or southern Italy [48], *Artemisia herba-alba* Asso [50]. In contrast, however, (+)-camphor was the dominant enantiomer in *A. arborescens* from Sicily [49] and *A. tridentata* subsp. *wyomingensis* and *A. tridentata* subsp. *vaseyana* from Idaho, USA [47]. Although (−)-borneol was the only enantiomer observed in *A. ludoviciana* (this work) and *A. tridentata* subsp. *wyomingensis* and subsp. *vaseyana* [47], (+)-borneol was the dominant enantiomer in *A. arborescens* [48].

### 2.3. Gutierrezia sarothrae

Six individual samples of *G. sarothrae* were collected and hydrodistillation of the aerial parts of the plants gave pale-yellow essential oils in yields ranging from 3.681% to 4.606%. The essential oils were analyzed by GC-MS and GC-FID (Table 6). The most abundant components in the *G. sarothrae* essential oils were the monoterpene hydrocarbons (*Z*)-β-ocimene (18.8–39.4%), limonene (1.4–25.4%), β-pinene (0.5–18.4%), and α-phellandrene (2.2–11.8%), along with the diacetylenes (*Z*,*E*)-matricaria ester (0.2–9.3%) and (*E*,*Z*)-matricaria ester (0.1–7.5%). There were also several unidentified components with relatively high concentrations in the *G. sarothrae* essential oils. The mass spectra of the major unidentified compounds are available as supplementary material (Supplementary Figure S2). Although present in small amounts, the presence of nepetalactones was unexpected.

A previous analysis of *G. sarothrae* essential oil from New Mexico reported geraniol (53.8%), γ-humulene (12.2%), *trans*-verbenol (6.0%), and verbenone (4.2%) as major components [35]. A subsequent examination of *G. sarothrae* essential oil from Utah by Epstein and Seidel [36] showed the major components to be (+)-α-pinene (12.6–22.9%), (−)-β-pinene (27.6–40.4%), (+)-limonene (7.2–13.1%), camphor (0.7–10.9%), (−)-pinocarvone (trace-11.3%), and (+)-verbenone (trace-6.0%). Another sample of *G. sarothrae* from New Mexico showed α-pinene (0.4–9.4%), β-pinene 0.7–9.6%), *p*-cymene (labeled as *o*-cymene, but the RI is more consistent with *p*-cymene, 2.5–7.9%), limonene (2.4–13.4%), cryptone (2.4–8.1%), bornyl acetate (2.8–4.5%), (*E*)-β-caryophyllene (2.3–4.8%), and β-eudesmol (0.1–5.9%) [37]. There is apparently much variation in the essential oil compositions of this plant and is likely due to subspecies variation [36]. Lane [27] has concluded, based on morphological characteristics, that “*Gutierrezia sarothrae* is an extremely variable taxon that possibly should be subdivided into a number of taxonomic varieties.” Ralphs and McDaniels have characterized eight chemotypes of *G. sarothrae* based on diterpenoid composition [30]. World Flora Online currently lists three varieties of *G. sarothrae* (var. *sarothrae*, var. *pomariensis* S.L. Welsh, and var. *pauciflora* Eastw.) [51].

The enantiomeric distributions of chiral terpenoid components were determined using chiral GC-MS (Table 7). (−)-α-Pinene (62.4–96.2%), (−)-β-pinene (97.3–99.8%), (−)-terpinen-4-ol (64.2–69.6%), (−)-α-terpineol (70.8–98.1%), and (−)-citronellol (64.7–70.2%) were the dominant enantiomers. Five of the six *G. sarothrae* essential oils showed (+)-limonene to be the major enantiomer (>90%), but sample *G.s.* #2 had (−)-limonene with 71.5%. Only one peak was observed for α-phellandrene, and its calculated RI was between the database RI values for (+)- and (−)-α-phellandrene, so identification of the enantiomer is in doubt. The predominance of (−)-β-pinene and (+)-limonene is in agreement with Epstein and Seidel [36]. In contrast, however, (−)-α-pinene was the major enantiomer in this present study, while Epstein and Seidel isolated (+)-α-pinene.

## 3. Materials and Methods

### 3.1. Plant Material, Hydrodistillation

Aerial parts of several individuals of *A. acanthicarpa*, *A. ludoviciana*, and *G. sarothrae* were collected from the Owyhee mountains of southwestern Idaho. The plants were identified by W.N. Setzer by comparison with samples from the New York Botanical Garden [52,53,54] and the Brigham Young University Herbarium via the Intermountain Region Herbarium Network [55]. Voucher specimens (WNS-Aa-7768, WNS-Al-7669, WNS-Al-7782, WNS-Gs-7772) have been deposited with the University of Alabama in Huntsville herbarium. The plant materials were frozen fresh (−20 °C) and stored frozen until distilled. For each plant sample, the fresh-frozen aerial parts were hydrodistilled for 4 h using a Likens-Nickerson apparatus with continuous extraction of the distillate with dichloromethane. The collection and hydrodistillation details are summarized in Table 8.

### 3.2. Gas Chromatographic Analyses

The essential oils of the aerial parts of *Ambrosia acanthicarpa*, *Artemisia ludoviciana*, and *Gutierrezia sarothrae* were analyzed by gas chromatography coupled with flame ionization detection (GC-FID, gas chromatography–mass spectrometry (GC-MS), and chiral GC-MS as previously described [56]. Instrumental details are provided as supplementary material (Supplementary Table S1). Retention indices (RI) were calculated based on a homologous series of *n*-alkanes using the linear equation of van den Dool and Kratz [57]. The essential oil components were identified by comparing their RI values (within ten RI units) and their MS fragmentation patterns (>80% similarity) with those reported in the Adams [58], FFNSC3 [59], NIST20 [60], and Satyal [61] databases. The compound percentages were based on raw peak areas without standardization. The individual enantiomers were determined from the chiral GC-MS analysis by comparison of RI values with authentic samples (Sigma-Aldrich, Milwaukee, WI, USA), which have been compiled in our in-house database. Percentages of each enantiomer were calculated from raw peak integration.

### 3.3. Hierarchical Cluster Analysis

The agglomerative hierarchical cluster (AHC) analysis was carried out on the *A. ludoviciana* essential oils using XLSTAT v. 2018.1.1.62926 (Addinsoft, Paris, France). The AHC analysis was performed using the concentrations of the 15 most abundant components (santolina triene, α-pinene, camphene, β-pinene, 1,8-cineole, lavender lactone, artemisia ketone, linalool, nonanal, camphor, borneol, terpinen-4-ol, α-terpineol, carvacrol, and davanone) from this current work as well as those previously reported compositions from the literature [21,22,23,24,25,26]. Dissimilarity was used to determine clusters, considering Euclidean distance, and Ward’s method was used to define agglomeration.

## 4. Conclusions

This is the first report on the chemical characterization of *A. acanthicarpa* essential oil. This species is wide-ranging in western North America, but the plants in this investigation were obtained from only one location in southwestern Idaho. Clearly, additional collections are needed to characterize the essential oil of this species more fully. In addition, this work complements previous investigations of *A. ludoviciana* by extending the geographical sampling as well as including enantiomeric distributions of chiral terpenoid components. It is apparent that not only the essential oil compositions, but also the enantiomeric distributions, are highly variable in *A. ludoviciana*. A comparison of essential oil analyses of *G. sarothrae* from this work and from previous investigations has revealed much variation in composition. Obviously, additional work on the essential oils of *A. acanthicarpa*, *A. ludoviciana*, and *G. sarothrae* are needed from different geographical locations. DNA barcode investigations may help to correlate with chemotypes of these species to help define the subspecies in these plants.

## Figures and Tables

**Table 1:** Chemical composition (percent of total) of the essential oil from the aerial parts of *Ambrosia acanthicarpa* from southwestern Idaho.

RI_calc_	RI_db_	Compounds	*A.a.* #1	*A.a.* #2	*A.a.* #3
842	840	1,2,5,5-Tetramethyl-1,3-cyclopentadiene	0.1	-	-
851	849	(2*E*)-Hexenal	0.1	0.1	tr
902	902	Santolina triene	1.7	0.5	0.8
923	923	Tricyclene	0.1	tr	0.1
925	925	α-Thujene	0.2	0.2	0.3
933	933	α-Pinene	36.7	38.2	45.1
949	950	Camphene	0.7	0.4	0.6
972	972	Sabinene	2.3	2.7	2.7
977	978	β-Pinene	0.8	0.9	0.9
984	984	6-Methylhept-5-en-2-one	tr	tr	0.1
989	989	Myrcene	22.4	25.5	21.6
991	996	3,3-Dimethyl-6-methylenecyclohexene	0.1	-	-
994	994	Yomogi alcohol	tr	tr	tr
1002	1002	Isobutyl 2-methylbutyrate	tr	tr	tr
1005	1004	*p*-Mentha-1(7),8-diene	0.7	0.8	0.6
1007	1006	α-Phellandrene	0.1	0.3	0.2
1009	1008	δ-3-Carene	tr	tr	tr
1017	1017	α-Terpinene	tr	tr	tr
1025	1025	*p*-Cymene	0.1	0.1	0.1
1029	1030	Limonene	3.2	4.7	3.0
1031	1031	β-Phellandrene	5.7	7.0	4.9
1032	1032	1,8-Cineole	0.2	0.3	0.2
1035	1034	(*Z*)-β-Ocimene	0.1	0.1	0.1
1045	1045	(*E*)-β-Ocimene	0.6	0.5	0.8
1050	1047	Santolina epoxide	0.1	tr	tr
1053	1053	Isobutyl angelate	0.1	tr	tr
1057	1057	γ-Terpinene	0.1	0.1	0.1
1070	1069	*cis*-Sabinene hydrate	0.1	0.1	0.1
1072	1072	*p*-Cresol	tr	tr	tr
1078	1079	Artemisia alcohol	tr	tr	tr
1084	1086	Terpinolene	0.1	0.1	0.1
1090	1093	Isobutyl tiglate	0.1	tr	tr
1099	1101	Linalool	0.1	tr	tr
1101	1103	Filifolone	0.3	tr	0.1
1102	1103	Isoamyl 2-methylbutyrate	tr	0.1	tr
1108	1107	2,6-Dimethylphenol	0.9	-	0.2
1108	1109	2-Methylbutyl isovalerate	-	0.1	-
1112	1113	(*E*)-4,8-Dimethylnona-1,3,7-triene	-	tr	tr
1122	1122	Chrysanthenone	1.6	0.1	0.5
1127	1125	α-Campholenal	0.1	tr	tr
1138	1138	*epi*-Photocitral A	-	-	tr
1141	1141	*trans*-Pinocarveol	tr	tr	-
1146	1145	*trans*-Verbenol	0.1	tr	tr
1148	1148	*trans*-Chrysanthemol	0.1	tr	0.1
1149	1149	Isobutyl hexanoate	-	tr	-
1152	1151	Citronellal	0.5	0.1	0.1
1154	1155	*cis*-Chrysenthemol	0.2	0.1	0.2
1157	1150	2-Methylbutyl angelate	0.1	0.1	0.1
1163	1165	Lavandulol	0.7	0.2	0.3
1170	1167	2-*exo*-Acetoxydihydrocamphene	tr	tr	-
1173	1170	Borneol	2.2	0.6	0.5
1177	1179	*iso*-Geranial	tr	0.1	0.1
1180	1180	Terpinen-4-ol	0.1	0.2	0.1
1190	1193	3-(2-Hydroxy-3-methyl-3-butenyl)-2,2-dimethyloxirane	0.1	tr	tr
1193	1195	2-Methylbutyl tiglate	0.1	tr	tr
1195	1195	α-Terpineol	0.2	0.1	0.1
1215	1217	Coumaran	-	tr	-
1228	1229	Thymyl methyl ether	-	tr	0.1
1238	1238	Neral	0.3	0.1	0.2
1249	1249	Geraniol	0.4	tr	-
1252	1252	Isoamyl hexanoate	0.1	0.1	-
1265	1266	*cis*-Chrysanthenyl acetate	tr	tr	tr
1268	1268	Geranial	0.3	0.1	0.2
1270	1270	*iso*-Piperitenone	0.1	-	tr
1276	1266	Chrysanthemyl acetate	tr	tr	0.1
1282	1284	Lavandulyl acetate	tr	tr	tr
1285	1286	Cogeijerene	0.7	1.3	0.6
1309	1309	4-Vinylguaicol	0.5	0.4	0.1
1311	1310	(*Z*)-Patchenol	tr	-	-
1317	1316	Filifolide A	tr	-	-
1323	1325	Silphiperfol-5-ene	-	-	0.2
1330	1330	Bicycloelemene	-	0.1	0.1
1345	1349	7-*epi*-Silphiperfol-5-ene	0.1	tr	0.2
1348	1350	Citronellyl acetate	tr	tr	-
1351	1356	Eugenol	0.1	tr	tr
1372	1369	Lavandulyl propionate	0.1	tr	0.1
1374	1375	α-Copaene	0.1	0.1	0.1
1377	1378	Geranyl acetate	0.1	tr	tr
1380	1382	Modheph-2-ene	0.4	0.2	0.3
1385	1384	Methyl (*E*)-cinnamate	-	tr	-
1386	1387	β-Cubebene	tr	0.2	0.2
1387	1385	α-Isocomene	0.4	0.2	0.2
1388	1390	*trans*-β-Elemene	tr	tr	tr
1392	1392	(*Z*)-Jasmone	-	tr	0.1
1393	1392	2-Ethylidene-6-methyl-3,5-heptadienal	0.8	tr	0.2
1399	1403	Methyl eugenol	0.1	-	-
1404	1406	α-Gurjunene	tr	0.1	0.1
1410	1411	β-Isocomene	0.2	0.1	0.2
1413	1415	Lavandulyl isobutyrate	0.1	tr	0.1
1417	1417	(*E*)-β-Caryophyllene	0.6	0.5	0.4
1430	1432	*trans*-α-Bergamotene	0.1	tr	tr
1447	1447	Geranylacetone	0.1	tr	0.1
1451	1452	(*E*)-β-Farnesene	0.1	0.1	0.2
1455	1454	α-Humulene	0.1	0.2	0.1
1457	1448	Neryl propionate	0.1	tr	tr
1468	1468	Geranyl propionate	0.1	tr	tr
1472	1475	Selina-4,11-diene	0.1	0.1	tr
1474	1475	γ-Muurolene	-	-	tr
1480	1480	Germacrene D	1.3	2.0	1.5
1483	1483	*trans*-β-Bergamotene	0.1	0.1	0.1
1488	1489	β-Selinene	0.1	0.1	0.1
1489	1489	(*Z*,*E*)-α-Farnesene	-	0.4	0.4
1491	1490	γ-Amorphene	-	0.1	-
1494	1497	Bicyclogermacrene	0.3	1.1	1.2
1497	1497	α-Muurolene	tr	0.1	tr
1500	1501	Lavandulyl isovalerate	0.6	0.3	0.5
1502	1504	(*E*,*E*)-α-Farnesene	0.3	0.7	0.8
1506	1508	β-Bisabolene	2.5	2.5	3.4
1508	1511	β-Curcumene	tr	tr	tr
1512	1512	γ-Cadinene	-	0.1	tr
1512	1511	Sesquicineole	0.1	-	0.1
1516	1518	δ-Cadinene	0.1	0.3	0.1
1518	1518	Javanol isomer II	-	-	0.1
1522	1520	(*E*,*Z*)-Matricaria ester	-	0.2	-
1526	1527	(*Z*,*E*)-Matricaria ester	0.1	0.2	tr
1550	1550	Geranyl butyrate	0.2	0.2	0.1
1556	1555	7-*epi-cis*-Sesquisabinene hydrate	0.1	-	-
1557	1560	(*E*)-Nerolidol	0.1	-	-
1569	1572	Citronellyl isovalerate	0.1	-	-
1572	1570	Neryl 2-methylbutyrate	0.1	-	-
1579	1574	Germacra-1(10),5-dien-4β-ol	0.2	0.2	0.2
1584	1587	Caryophyllene oxide	0.2	0.1	0.1
1598	1596	Geranyl 2-methylbutyrate	0.2	0.1	0.1
1624	1625	Junenol	0.1	-	-
1644	1643	τ-Cadinol	0.2	0.3	0.2
1653	1652	Geranyl pentanoate	tr	-	-
1657	1655	α-Cadinol	0.1	0.3	0.1
1661	1661	*neo*-Intermedeol	0.1	0.1	0.1
1671	1671	β-Bisabolol	0.6	0.2	0.4
1687	1688	α-Bisabolol	-	0.1	-
1716	1716	Citronellyl hexanoate	0.1	-	-
1727	1730	δ-Dodecalactone	0.1	-	-
1749	1748	Geranyl hexanoate	0.2	0.1	-
1763	1762	*cis*-Lanceol	-	tr	tr
1840	1841	Phytone	-	0.1	tr
		Monoterpene hydrocarbons	75.5	82.2	81.7
		Oxygenated monoterpenoids	10.4	2.5	3.8
		Sesquiterpene hydrocarbons	6.9	9.3	9.7
		Oxygenated sesquiterpenoids	1.9	1.2	1.2
		Benzenoid aromatics	1.5	0.4	0.4
		Others	1.7	2.0	0.9
		Total identified	98.0	97.6	97.7

RI_calc_ = Retention index determined with respect to a homologous series of *n*-alkanes on a ZB-5ms column. RI_db_ = Reference retention index from the databases. *A.a.* = *Ambrosia acanthicarpa*. tr = trace (<0.05%). - = not detected.

**Table 2:** Major components of *Ambrosia* essential oils from the literature.

*Ambrosia* Species	Geographical Location	Major Components (>5%)	Ref.
*Ambrosia arborescens* Mill.	Cuzco, Peru	β-Acoradiene (15.3%), chrysanthenone (11.3%), germacrene D (7.6%), shyobunol (6.7%), β-cadinene (6.2%)	[40]
*Ambrosia artemisiifolia* L.	Bor, Serbia	Germacrene D (25.3%), limonene (21.6%), α-pinene (15.7%)	[41]
*Ambrosia artemisiifolia* L.	Xinyuan, China	Germacrene D (32.9%), β-pinene (15.1%), limonene (9.9%)	[42]
*Ambrosia confertiflora* DC.	North-central Israel	Chrysanthenone (25.0%), *cis*-chrysanthenol (17.7%), germacrene D (12.3%), (*E*)-β-caryophyllene (11.1%), (*E*)-β-ocimene (6.9%)	[43]
*Ambrosia cumanensis* Kunth	San Rafael, Costa Rica	Bicyclogermacrene (14.7–23.4%), germacrene D (10.1–16.9%), α-pinene (7.8–12.8%), chrysanthenone (6.2–8.7%), β-pinene (4.5–6.7%), limonene (3.5–5.8%)	[39]
*Ambrosia grayi* (A. Nelson) Shinners	Cultivated, Ramat Yishay, Israel	Myrcene (17.9%), germacrene D (17.8%), unidentified (15.4%), limonene (14.2%), borneol (11.3%), (*E*)-β-caryophyllene (10.8%)	[43]
*Ambrosia peruviana* Willd. (syn. *Ambrosia cumanensis* Kunth)	Los Operadores, Ecuador	γ-Curcumene (52.0%), chrysanthenone (5.6%), *ar*-curcumene (5.1%)	[44]
*Ambrosia tenuifolia* Spreng.	Ness-Zionia, Israel	Myrcene (32.8%), (2E)-hexenal (13.3%), germacrene D (7.2%), (*E*)-β-ocimene (5.4%)	[43]
*Ambrosia trifida* L.	Despotovo, Serbia	Limonene (20.7%), bornyl acetate (15.0%), borneol (14.7%), germacrene D (11.6%)	[45]

**Table 3:** Enantiomeric distribution of chiral terpenoids in the essential oil from the aerial parts of *Ambrosia acanthicarpa*.

Compounds	RI_db_	RI_calc_	*A.a.* #1	*A.a.* #2	*A.a.*#3
(+)-α-Thujene	950	951	22.3	22.1	26.2
(−)-α-Thujene	951	952	77.7	77.9	73.8
(−)-α-Pinene	976	971	99.3	99.4	99.4
(+)-α-Pinene	982	981	0.7	0.6	0.6
(−)-Camphene	998	1001	95.8	93.5	95.3
(+)-Camphene	1005	1005	4.2	6.5	4.7
(+)-Sabinene	1021	1021	53.7	48.3	48.2
(−)-Sabinene	1030	1029	46.3	51.7	51.8
(+)-β-Pinene	1027	1027	13.5	12.4	12.1
(−)-β-Pinene	1031	1032	86.5	87.6	87.9
(−)-α-Phellandrene	1050	-	-	0.0	0.0
(+)-α-Phellandrene	1053	1053	-	100.0	100.0
(−)-Limonene	1073	1074	60.4	50.2	56.9
(+)-Limonene	1081	1081	39.6	49.8	43.1
(−)-β-Phellandrene	1083	1084	2.0	1.8	3.0
(+)-β-Phellandrene	1089	1087	98.0	98.2	97.0
(−)-Lavandulol	1314	1316	100.0	100.0	100.0
(−)-Borneol	1335	1337	100.0	100.0	100.0
(+)-Borneol	1340				
(−)-α-Terpineol	1347	1347	86.7	-	-
(+)-α-Terpineol	1356	1356	13.3	-	-
(−)-(*E*)-β-Caryophyllene	1461	1461	100.0	100.0	100.0
(+)-Germacrene D	1519	1519	0.0	6.5	0.0
(−)-Germacrene D	1522	1522	100.0	93.5	100.0
(+)-β-Bisabolene	1546	1546	100.0	100.0	100.0
(−)-β-Bisabolene	1549	no	0.0	0.0	0.0
(−)-δ-Cadinene	1563	no	-	0.0	0.0
(+)-δ-Cadinene	1576	1576	-	100.0	100.0

RI_db_ = Retention index from our in-house database. RI_calc_ = Calculated retention index based on a homologous series of *n*-alkanes on a Restek B-Dex 325 capillary column. *A.a.* = *Ambrosia acanthicarpa*. no = not observed. - = compound not detected.

**Table 4:** Chemical composition (percent of total) of the essential oil from the aerial parts of *Artemisia ludoviciana* from the Owyhee Mountains of Idaho.

RI_calc_	RI_db_	Compounds	*A.l.* B1	*A.l.* B2	*A.l.* B3	*A.l.* C1	*A.l.* C2	*A.l.* T1	*A.l.* T2	*A.l.* T3	*A.l.* U1	*A.l.* U2	*A.l.* U3	*A.l.* U4
872	872	2-Methylbutyl acetate	0.2	0.1	0.2	0.6	0.8	0.3	0.1	0.7	0.4	0.3	1.1	0.9
902	902	Santolina triene	5.1	tr	0.1	18.8	12.1	0.2	1.9	0.1	tr	9.0	0.1	0.5
913	913	Isobutyl isobutyrate	0.3	0.1	tr	0.1	0.1	0.1	0.2	0.1	0.5	0.1	0.4	-
921	922	Artemisia triene	tr	-	tr	-	-	0.1	0.2	-	-	0.1	-	0.2
922	923	Tricyclene	-	0.4	-	-	0.2	0.1	0.4	-	0.6	-	-	-
924	925	α-Thujene	tr	0.2	tr	0.1	0.5	tr	tr	0.1	0.1	-	0.1	-
932	933	α-Pinene	0.1	3.2	0.1	0.2	0.7	0.7	2.9	0.9	0.6	0.1	0.4	1.3
936	935	Ethyl tiglate	-	-	-	-	-	-	-	-	0.1	0.1	-	-
947	945	4-Methyl pent-2-enolide	-	0.1	0.1	0.9	-	-	-	4.6	0.1	2.9	4.9	0.3
948	950	Camphene	0.1	6.1	0.1	-	1.7	2.2	8.0	-	7.5	-	-	0.1
962	964	Benzaldehyde	tr	tr	0.1	tr	tr	tr	-	0.1	tr	-	tr	tr
969	968	Isoamyl propionate	0.2	0.2	tr	0.5	0.2	tr	0.1	tr	0.3	-	0.2	tr
971	971	Sabinene	-	1.1	tr	-	-	0.5	tr	0.8	0.5	-	0.5	0.1
971	971	Artemiseole	4.0	-	-	11.4	11.2	-	3.0	-	-	4.5	-	-
977	978	β-Pinene	0.2	1.4	0.2	0.2	0.3	0.6	0.9	0.6	0.4	0.1	0.3	tr
984	984	6-Methyl-5-hepten-2-one	0.1	-	0.1	-	-	-	-	0.1	0.1	-	0.1	-
987	989	Myrcene	-	0.1	-	tr	tr	tr	-	tr	tr	-	0.1	-
989	990	Dehydro-1,8-cineole	-	0.3	-	0.1	-	0.3	0.1	0.8	0.3	-	0.4	-
989	989	*trans*-Dehydroxylinalool oxide	tr	-	-	-	0.2	-	-	-	0.1	-	-	-
990	990	*trans*-Dehydrolinalool oxide	-	-	-	-	-	-	-	-	-	-	0.3	0.1
990	---	(*E*)-2,6-Dimethyl-3,5-heptadien-2-ol	0.1	-	tr	-	-	-	-	-	-	-	-	0.1
994	994	Yomogi alcohol	0.2	-	1.0	0.3	0.2	8.5	tr	-	-	-	-	12.7
1000	---	Unidentified (RI 1000)	-	-	-	-	-	-	-	1.8	-	1.0	0.5	-
1002	1003	Isobutyl 2-methylbutyrate	0.3	0.1	tr	0.1	tr	0.1	0.1	0.1	0.4	-	tr	-
1005	1005	*cis*-Dehydroxylinalool oxide	0.1	-	-	-	0.1	-	-	-	-	-	-	-
1005	1005	(3*Z*)-Hexenyl acetate	-	tr	-	-	-	-	-	-	-	-	-	-
1006	1006	α-Phellandrene	-	-	-	-	-	-	0.1	0.3	0.2	-	-	-
1006	1006	*cis*-Dehydrolinalool oxide	-	-	-	-	-	-	-	0.2	0.2	-	0.3	0.1
1006	1005	Isobutyl isovalerate	0.2	tr	-	tr	-	tr	-	-	0.1	-	-	-
1009	---	Unidentified (RI 1009)	-	-	-	-	-	-	-	1.2	-	0.7	0.7	-
1010	1009	δ-3-Carene	-	-	-	-	-	-	-	tr	tr	-	-	-
1011	1012	Isoamyl isobutyrate	-	tr	-	tr	-	-	-	-	-	-	-	-
1014	1015	2-Methylbutyl isobutyrate	0.6	0.4	0.1	0.3	0.3	0.4	0.5	0.3	0.8	0.2	0.9	0.1
1016	---	Unidentified (RI 1016)	-	-	-	-	-	-	-	1.5	-	0.9	0.4	-
1017	1017	α-Terpinene	tr	0.5	-	0.1	0.2	0.4	0.3	1.4	0.8	0.1	0.7	tr
1022	1022	Ethyl 3-methylbut-3-enyl carbonate	-	-	-	-	-	-	-	tr	-	-	0.1	-
1025	1025	*p*-Cymene	-	0.2	-	tr	0.1	0.4	0.5	0.7	0.2	0.1	0.5	tr
1027	1026	2-Acetyl-3-methylfuran	-	0.1	-	-	-	0.2	-	-	-	-	-	-
1028	1030	Limonene	-	0.8	-	0.1	0.7	23.7	0.4	0.2	0.2	0.1	0.2	tr
1030	1031	β-Phellandrene	-	-	-	-	-	-	0.2	0.9	0.6	tr	0.3	-
1030	1031	Santolina alcohol	-	-	0.1	tr	tr	-	-	-	-	-	-	0.1
1032	1032	1,8-Cineole	1.1	26.0	0.7	6.7	3.9	0.1	4.6	50.8	21.8	2.5	20.9	2.0
1034	1034	(*Z*)-β-Ocimene	-	-	-	0.2	-	-	-	-	-	-	-	-
1037	1035	Lavender lactone	0.1	0.1	0.2	0.2	0.1	-	-	0.1	0.1	tr	1.1	0.3
1039	1040	Butyl 2-methylbutyrate	-	tr	-	-	-	-	-	tr	tr	-	-	-
1045	1045	Phenylacetaldehyde	0.3	0.1	0.1	-	-	tr	tr	0.1	0.1	0.2	0.2	0.2
1045	1045	(*E*)-β-Ocimene	-	-	-	0.1	-	-	-	-	-	-	-	-
1049	1047	Santolina epoxide	1.9	-	-	4.7	6.2	-	0.3	0.4	-	1.2	-	-
1049	1049	*cis*-Arbusculone	-	0.2	tr	0.2	-	-	-	tr	-	-	1.1	-
1050	1051	2,3,6-Trimethylhepta-1,5-diene	0.1	-	0.2	-	-	-	-	tr	-	-	-	-
1053	1053	Bergamal	-	-	-	-	-	-	-	-	-	0.1	-	-
1053	1050	Prenyl isobutyrate	-	tr	-	-	-	-	-	-	-	-	-	-
1057	1056	Artemisia ketone	-	-	14.5	-	-	0.8	-	0.2	-	-	-	46.1
1057	1057	γ-Terpinene	tr	0.9	-	0.3	0.4	0.7	0.4	1.7	0.9	0.1	0.8	-
1061	1059	Verbenone	-	-	-	-	-	-	-	-	-	0.2	-	-
1068	1068	*trans*-Arbusculone	-	0.2	-	0.6	-	-	-	-	-	-	1.5	-
1069	1067	*cis*-Linalool oxide (furanoid)	0.5	-	0.2	-	1.0	-	-	0.1	0.1	-	-	0.3
1069	1070	4-Nonanone	-	-	-	-	-	-	-	-	-	0.2	-	-
1069	1069	*cis*-Sabinene hydrate	-	0.8	-	-	-	6.7	-	tr	tr	-	-	-
1071	1072	α-Santolina alcohol	0.2	-	-	0.5	0.4	-	0.2	0.5	-	1.0	0.4	-
1078	1079	Artemisia alcohol	-	-	3.6	0.2	0.1	0.3	-	-	-	-	-	7.7
1084	1086	Terpinolene	-	0.2	-	0.1	0.1	0.2	0.1	0.4	0.3	0.1	0.3	-
1085	1086	*trans*-Linalool oxide (furanoid)	0.3	-	0.1	-	0.4	0.2	-	-	0.1	-	-	0.2
1089	1089	Isoamyl phenylacetate	-	-	-	-	-	-	-	-	tr	-	-	-
1090	1093	Phenethyl isovalerate	-	-	-	-	-	-	-	-	0.1	-	-	-
1090	1091	*p*-Cymenene	-	-	-	-	-	-	-	-	0.1	-	-	-
1101	1101	Linalool	15.6	0.1	2.1	0.3	19.9	0.7	0.2	0.9	1.1	0.6	6.1	1.6
1101	1103	Filifolone	-	-	-	-	-	-	-	-	0.4	-	-	-
1102	1101	*trans*-Sabinene hydrate	-	-	-	-	-	0.4	-	-	-	-	-	-
1102	1103	2-Methylbutyl 3-methylbutyrate	0.8	1.5	0.3	0.3	0.5	0.1	0.2	0.6	1.1	0.3	0.4	0.1
1103	1102	6-Methylhepta-3,5-dien-2-one	-	-	0.1	-	-	-	0.1	-	-	0.2	-	-
1104	1104	Hotrienol	0.4	-	-	-	0.9	-	-	0.6	0.5	-	0.6	0.8
1104	1104	Nonanal	-	0.1	0.1	0.1	0.1	-	0.1	0.2	0.1	0.1	0.1	0.1
1107	1103	Tetrahydromyrcenol	-	-	-	-	-	-	-	-	-	-	-	0.2
1107	1108	Amyl isovalerate	0.7	0.1	0.1	0.1	-	0.1	tr	0.2	0.3	0.1	-	-
1109	1110	α-Thujone	-	-	-	-	-	-	-	-	-	-	1.0	-
1110	1110	3-Methyl-3-butenyl 3-methylbutyrate	-	tr	-	-	-	-	-	0.1	-	-	-	-
1113	1113	1,3,8-*p*-Menthatriene	-	-	-	-	-	-	-	tr	-	-	-	-
1119	1118	β-Thujone	-	-	-	-	-	-	-	-	-	-	0.1	-
1121	1122	Dehydrosabina ketone	-	-	-	-	-	-	-	tr	-	-	-	-
1122	1122	Chrysanthenone	-	-	-	-	-	0.1	-	tr	0.6	-	-	-
1122	1122	*trans-p*-Mentha-2,8-dien-1-ol	-	0.1	-	-	-	-	-	-	-	-	-	-
1124	1124	*cis-p*-Menth-2-en-1-ol	-	0.1	-	-	-	0.2	-	tr	-	-	-	-
1127	1127	α-Campholenal	-	0.1	-	-	-	0.1	0.1	-	tr	-	-	-
1130	1130	Cuminaldehyde	-	-	-	-	-	-	-	-	-	0.1	-	-
1132	1131	Limona ketone	-	-	-	-	-	-	-	tr	-	-	-	-
1132	---	Unidentified (RI 1132)	-	-	-	-	1.3	-	-	-	-	-	-	-
1132	1132	Butyl tiglate	-	tr	-	-	-	-	-	-	-	-	-	-
1137	1137	*cis-p*-Mentha-2,8-dien-1-ol	-	0.1	-	-	-	-	0.5	-	-	-	-	-
1137	---	*neo*-Lyratol	1.3	-	0.1	4.2	4.3	-	0.1	-	-	22.0	-	-
1141	1141	*trans*-Pinocarveol	-	-	-	-	-	-	-	0.1	-	-	-	-
1144	1142	*trans-p*-Menth-2-en-1-ol	-	0.1	-	-	-	0.2	-	tr	-	-	-	-
1144	1145	*trans*-Verbenol	-	-	-	tr	0.1	-	-	-	-	-	-	-
1145	1147	Nerol	-	-	-	-	-	-	-	-	-	-	-	0.2
1149	1149	Camphor	0.3	30.7	0.2	0.1	9.5	13.6	61.9	0.4	49.0	0.8	1.1	1.2
1151	1152	Nerol oxide	-	-	-	-	-	-	-	0.1	-	-	-	-
1154	1156	Lyratol	-	-	-	0.9	0.8	-	-	-	-	1.1	-	-
1156	1155	*cis*-Chrysanthemol	14.2	-	3.0	0.3	1.0	0.2	0.9	-	-	-	-	-
1162	1162	β-Artemisyl acetate	-	-	0.3	-	-	6.9	-	-	-	-	-	4.8
1163	1164	Pinocarvone	-	0.2	-	-	-	-	0.3	0.1	0.2	-	-	-
1164	1166	*cis*-Chrysanthenol	-	-	-	-	-	0.2	-	-	-	-	-	-
1164	1165	*iso*-Borneol	-	-	-	-	-	-	-	-	tr	-	-	-
1164	1165	Lavandulol	-	-	-	0.3	0.4	-	-	-	-	-	-	-
1164	1164	Pinocarvone	-	-	-	-	-	-	-	-	-	-	0.1	-
1170	1170	δ-Terpineol	-	0.5	-	0.1	-	0.5	0.1	0.7	0.3	-	0.2	-
1172	1171	*p*-Mentha-1,5-dien-8-ol	-	-	-	-	-	-	-	0.1	-	-	-	-
1172	1173	Borneol	-	2.1	-	-	0.2	4.3	2.9	-	1.4	-	-	-
1180	1180	Terpinen-4-ol	0.1	2.7	0.1	0.6	1.0	2.6	1.0	4.0	1.7	0.2	1.6	0.2
1186	---	Unidentified (RI 1186)	-	-	1.0	-	-	-	-	-	-	-	-	1.2
1187	1186	*p*-Cymen-8-ol	-	-	-	-	-	tr	-	-	-	-	-	-
1188	1188	(3*Z*)-Hexenyl butyrate	0.1	-	-	-	0.2	-	-	-	-	-	-	0.1
1189	1189	Geraniol	0.1	-	0.1	-	-	-	-	-	-	-	-	-
1192	1192	Methyl salicylate	0.1	-	-	0.1	-	tr	-	-	-	-	-	-
1193	1192	2-Methylbutyl tiglate	0.1	0.1	-	-	-	-	0.1	-	-	-	-	-
1195	1195	α-Terpineol	0.2	2.3	0.1	0.6	0.5	2.1	0.4	1.5	0.7	0.1	0.8	0.1
1197	1197	Myrtenal	-	-	-	-	-	-	0.1	-	-	-	-	-
1199	1197	Lilac aldehyde B	-	-	-	-	-	-	-	-	-	-	0.2	-
1200	1197	Lilac aldehyde C	-	-	-	-	-	-	-	-	-	-	0.2	-
1208	1208	Verbenone	-	-	-	-	0.2	0.2	tr	0.3	-	-	-	-
1211	---	Unidentified (RI 1211)	-	-	-	-	-	-	-	-	-	-	-	1.0
1232	1232	*cis*-Carveol	-	-	-	-	-	1.1	-	-	-	-	-	-
1236	---	Unidentified (RI 1236)	-	-	1.2	0.2	0.3	-	-	-	-	0.5	-	2.9
1238	1238	Neral	-	-	-	-	-	tr	0.1	0.1	-	-	-	-
1239	1240	Ascaridole	-	-	-	-	-	tr	-	-	-	-	-	-
1240	1241	Pulegone	-	-	-	-	-	-	-	-	-	-	-	0.3
1245	1246	Carvone	-	-	-	-	-	0.2	-	-	0.1	-	-	-
1247	1247	*trans*-Chrysanthenyl acetate	0.2	-	-	0.9	1.4	-	-	-	-	16.1	0.2	0.1
1252	1252	Chavicol	-	-	-	-	-	-	-	-	tr	-	-	0.1
1254	1254	2-Phenethyl acetate	-	-	-	-	-	tr	-	-	tr	-	-	-
1265	1266	*cis*-Chrysanthenyl acetate	0.1	-	-	0.3	0.7	0.7	-	0.1	-	0.6	-	-
1268	1268	Geranial	-	-	-	-	-	0.1	0.1	0.1	-	-	0.1	-
1270	1270	*iso*-Piperitenone	-	-	-	-	-	0.1	-	-	0.2	-	-	-
1274	1273	Methyl hydrocinnamate	-	-	-	-	-	-	-	0.1	-	-	-	-
1275	---	Chrysanthemyl acetate	1.2	-	4.4	0.1	0.2	2.6	0.3	-	-	-	-	0.1
1282	1282	Bornyl acetate	tr	5.0	tr	-	0.2	1.6	0.3	-	1.5	-	0.3	0.1
1282	1284	Lavandulyl acetate	0.1	-	0.6	0.2	0.4	0.1	tr	-	-	0.4	-	0.3
1288	1291	*trans*-Sabinyl acetate	-	-	-	-	-	-	-	-	-	-	0.1	-
1288	1289	*trans*-Verbenyl acetate	-	-	-	-	-	0.2	0.1	-	-	-	-	-
1289	1289	Thymol	-	-	-	-	-	-	0.1	-	-	-	-	-
1297	1296	Carvacrol	-	-	-	-	-	-	tr	-	-	-	-	-
1305	1306	*iso*-Ascaridole	-	-	-	-	-	0.1	0.1	-	-	-	-	-
1311	1313	δ-Terpinyl acetate	-	0.1	-	-	-	-	-	0.1	0.1	-	0.1	-
1320	1325	(3*Z*)-Hexenyl tiglate	-	0.1	-	-	-	-	-	-	-	-	-	-
1332	1332	*trans*-Carvyl acetate	-	-	-	-	-	0.1	-	-	-	-	-	-
1345	1346	α-Terpinyl acetate	-	0.5	-	0.1	0.1	-	-	0.3	0.3	-	0.3	-
1347	1347	Ethyl hydrocinnamate	-	-	-	-	-	-	-	0.1	-	-	-	-
1352	1356	Eugenol	0.1	0.1	-	0.5	0.1	0.1	-	-	0.3	-	-	0.4
1355	1356	*p*-Acetanisole	-	-	-	-	-	-	-	0.1	-	-	-	-
1357	1361	Neryl acetate	-	tr	-	-	-	-	-	0.1	-	-	0.1	-
1358	1357	*cis*-Carvyl acetate	-	-	-	-	-	0.9	-	-	-	-	-	-
1373	1375	α-Copaene	0.3	tr	0.3	0.6	0.1	0.1	tr	0.1	tr	0.2	0.1	0.1
1377	1378	Geranyl acetate	-	0.1	-	-	-	tr	-	0.4	tr	-	0.8	-
1378	1379	(*E*)-β-Damascenone	-	-	-	-	-	tr	-	-	-	-	-	-
1382	1382	β-Bourbonene	-	-	-	-	-	tr	-	-	-	-	-	-
1389	1390	*trans*-β-Elemene	-	-	-	-	-	-	-	-	-	0.2	-	-
1393	1394	(*Z*)-Jasmone	-	-	0.1	0.1	0.1	-	0.1	0.2	0.1	0.3	0.4	0.1
1398	1396	(2*E*)-1,3,7-Trimethyl-2,6-octadienyl acetate	0.2	-	1.5	-	0.1	4.4	0.5	-	-	-	-	0.1
1416	---	Unidentified (RI 1416)	-	-	0.5	-	-	3.6	-	-	-	-	0.1	0.1
1418	1417	(*E*)-β-Caryophyllene	-	-	-	0.2	0.1	-	-	-	0.1	0.2	0.1	0.1
1428	1427	γ-Elemene	-	-	-	-	-	-	-	-	-	0.1	-	-
1456	1460	Cabreuva oxide B	-	-	0.1	-	-	-	-	-	-	-	-	-
1458	1457	Valerana-7,11-diene	-	-	-	-	-	-	-	-	-	0.2	-	-
1462	---	Unidentified (RI 1462)	0.4	-	0.5	0.2	0.4	-	0.3	-	-	2.4	0.3	0.2
1463	1463	γ-Decalactone	tr	tr	0.1	0.1	-	0.1	tr	0.1	tr	0.2	0.1	-
1467	1468	Geranyl propionate	-	-	-	-	-	-	-	-	-	-	0.2	-
1470	1472	*trans*-Cadina-1(6),4-diene	-	-	-	0.1	-	-	-	-	-	-	-	-
1472	1474	Amorpha-4,7(11)-diene	-	-	-	-	-	-	-	-	-	0.2	-	-
1472	1475	Selina-4,11-diene	-	-	-	-	-	-	0.1	-	0.1	0.3	-	-
1477	1479	γ-Curcumene	-	-	-	-	-	-	-	-	-	-	-	0.1
1479	1480	Germacrene D	0.4	0.3	0.4	0.9	0.4	0.4	tr	0.2	tr	0.6	-	0.5
1479	1480	*ar*-Curcumene	-	-	-	-	-	-	-	0.1	0.1	-	0.1	-
1480	1483	Davana ether 1	-	0.1	-	0.3	-	-	-	-	-	-	-	-
1482	1478	γ-Muurolene	-	-	-	-	-	-	-	-	-	0.2	-	-
1483	1483	Phenethyl 2-methylbutyrate	0.1	0.1	tr	-	-	tr	-	-	0.1	-	-	-
1486	1488	δ-Selinene	-	-	-	-	-	-	-	-	-	1.1	0.1	-
1486	1491	Eremophilene	-	-	-	0.3	0.2	-	-	0.1	-	-	-	-
1487	1489	β-Selinene	-	-	0.2	-	-	-	0.1	0.1	tr	0.2	0.2	-
1488	1490	(*Z*)-Jasmin lactone	-	-	0.2	0.1	-	0.1	-	-	-	0.2	0.1	tr
1488	1493	Phenethyl isovalerate	0.2	-	-	-	-	-	-	-	-	-	-	-
1489	1489	Isoamyl phenylacetate	-	-	-	-	-	-	-	0.1	-	-	-	0.1
1490	1492	Valencene	-	-	-	-	-	-	-	0.1	-	0.1	-	-
1490	1490	γ-Amorphene	-	-	-	0.2	-	-	-	-	-	-	-	tr
1493	1497	Bicyclogermacrene	-	0.1	0.1	-	-	tr	-	-	-	-	-	0.1
1494	1497	*epi*-Cubebol	-	-	-	0.3	-	-	-	-	-	-	-	-
1495	1500	Chrysanthemyl 2-methylbutyrate	0.1	-	-	-	-	-	-	-	-	-	-	-
1495	1497	α-Selinene	-	-	-	-	-	-	-	-	0.1	-	-	-
1496	1500	α-Muurolene	0.1	-	0.2	0.2	-	-	-	0.1	tr	0.1	0.1	tr
1501	1502	Davana ether 2	-	0.2	-	0.8	-	-	-	-	-	-	-	0.1
1502	1504	Davana ether 3	-	tr	-	0.2	-	-	-	-	-	-	-	-
1511	1512	γ-Cadinene	-	tr	-	0.1	-	tr	-	0.2	-	-	-	-
1514	1516	Artedouglasia oxide C	-	0.5	-	2.5	-	-	-	0.2	-	-	5.0	-
1515	1515	Cubebol	-	-	-	-	-	-	-	-	-	-	-	tr
1515	1518	δ-Cadinene	0.2	tr	0.2	0.5	0.1	0.1	tr	-	-	0.2	-	0.3
1520	1519	*trans*-Calamenene	-	-	0.1	-	-	-	-	-	-	0.1	-	-
1520	1521	Davana ether 4	-	0.1	-	0.6	-	-	-	-	-	-	-	tr
1522	1521	Zonarene	-	-	-	-	-	-	-	-	-	0.1	-	-
1524	1526	Laciniata furanone G	-	-	-	0.1	-	-	-	-	-	-	0.6	-
1528	1528	Artedouglasia oxide A	-	0.8	-	3.0	-	-	-	0.2	-	-	6.8	0.1
1536	1536	Laciniata furanone F	-	-	-	0.1	-	-	-	-	-	-	0.7	-
1539	1539	Laciniata furanone E	-	-	-	0.2	-	-	-	-	-	-	0.9	-
1548	1546	α-Elemol	-	-	-	-	-	-	-	0.3	-	0.9	-	-
1548	1549	Davanone B	-	0.7	-	2.9	0.1	-	-	-	-	-	2.6	0.2
1550	1549	Laciniata furanone H	-	0.3	-	1.1	-	-	-	0.2	-	-	2.3	tr
1554	1555	(*Z*)-Dihydronerolidol	-	-	0.7	-	-	-	-	-	-	-	-	-
1554	1556	Davanone C	-	0.7	-	3.0	0.1	-	-	-	-	-	2.1	0.1
1561	1560	(*E*)-Nerolidol	35.1	0.5	41.0	0.3	3.3	0.1	0.2	0.1	-	-	0.4	0.1
1561	1562	Davanone D	-	0.6	-	1.7	-	-	-	-	-	-	1.5	0.2
1569	1570	(*E*)-Dihydronerolidol	0.6	-	1.2	-	-	-	-	-	-	-	-	-
1573	1573	Artedouglasia oxide B	-	0.4	-	1.6	-	-	-	-	-	-	2.9	-
1577	1577	Davanone	-	0.4	0.2	1.6	0.1	-	-	-	-	-	2.8	-
1578	1574	Germacra-1(10),5-dien-4β-ol	-	-	-	-	-	-	-	0.7	tr	1.1	-	0.3
1582	1583	Phenethyl tiglate	-	tr	-	-	-	tr	-	-	-	-	-	-
1582	1587	Caryophyllene oxide	-	-	-	0.5	-	0.1	-	-	-	-	-	0.3
1590	1590	Globulol	-	-	0.2	0.1	-	-	-	-	-	-	-	-
1592	1593	Salvial-4(14)-en-1-one	-	-	-	0.1	-	-	-	-	-	-	-	-
1594	1594	Viridiflorol	-	-	-	0.1	-	-	-	-	-	-	-	-
1595	1596	Fokienol	1.5	0.1	1.6	0.3	0.7	-	0.2	-	-	-	-	0.3
1598	---	Unidentified (RI 1598)	-	-	-	0.1	-	-	-	0.8	tr	1.3	1.1	-
1602	1600	α-Oplopenone	-	-	-	0.5	0.1	-	-	-	-	-	-	-
1604	1605	Ledol	-	-	0.1	-	-	-	-	-	-	-	-	-
1605	---	Isoamyl 3-phenylpropionate	-	-	-	-	-	-	-	0.2	-	-	-	-
1606	1605	Davanone E	-	-	-	-	-	-	0.1	-	-	-	-	-
1610	1611	Germacra-1(10),5-dien-4α-ol	-	-	-	0.1	-	-	-	1.4	0.1	2.1	1.8	-
1615	1612	5-*epi*-7-*epi*-β-Eudesmol	-	-	-	-	-	-	-	-	-	0.2	-	-
1619	---	Unidentified (RI 1619)	0.6	-	1.1	0.2	0.6	-	0.2	-	0.2	-	-	0.8
1627	1628	1-*epi*-Cubenol	-	-	-	0.3	-	-	-	-	-	-	-	0.1
1628	1628	Methyl (*E*)-jasmonate	-	-	-	-	-	-	tr	-	-	-	-	-
1630	1632	γ-Eudesmol	-	0.2	-	-	-	-	-	-	-	-	-	-
1631	1631	Eremoligenol	-	-	-	-	-	-	-	1.8	-	0.4	-	-
1633	1630	Caryophylla-4(12),8(13)-dien-5α-ol	0.4	0.1	0.6	0.1	0.3	-	0.2	-	0.1	-	-	0.5
1634	1632	γ-Eudesmol	-	-	-	-	-	-	-	-	-	2.7	-	-
1641	1642	Methyl (*Z*)-jasmonate	0.6	0.4	1.1	0.9	0.6	0.6	0.6	0.7	0.2	0.7	1.8	0.5
1643	1644	τ-Muurolol	0.2	-	0.3	0.2	-	-	-	-	-	-	-	0.1
1645	1645	α-Muurolol (=δ-Cadinol)	0.2	-	0.2	-	-	-	-	-	-	-	-	-
1654	---	Unidentified (RI 1654)	-	-	1.1	-	-	-	-	-	-	-	-	-
1655	1655	α-Cadinol	0.6	0.1	-	0.5	0.3	0.1	-	-	-	-	-	0.1
1655	1656	β-Eudesmol	-	-	-	-	-	-	-	0.4	-	0.6	-	-
1658	1660	Selin-11-en-4α-ol	-	-	-	-	-	0.9	-	-	-	-	-	0.3
1671	---	Unidentified (RI 1671)	0.8	-	1.8	-	0.4	-	-	-	-	-	-	0.1
1673	1673	Methyl (*E*)-*epi*-jasmonate	0.4	0.1	1.2	0.2	0.3	0.3	0.3	0.3	0.1	0.3	0.6	0.2
1676	1674	γ-Dodecalactone	-	-	-	-	-	tr	tr	0.1	-	-	-	-
1687	---	Unidentified (RI 1687)	-	-	-	-	-	-	-	6.8	-	6.5	5.1	1.0
1726	1722	(2*E*,6*E*)-Farnesol	1.3	-	2.8	-	-	-	-	-	-	-	-	-
1780	---	Unidentified (RI 1780)	-	2.2	-	5.7	-	-	-	-	-	-	-	0.3
1945	---	Gazaniolide	-	-	-	0.4	0.2	-	-	-	-	-	-	0.2
2129	2131	(3*Z*,6*Z*)-9,10-Epoxynonadecadiene	0.4	-	0.8	-	-	-	-	-	-	-	-	-
2142	2143	Serratol	0.7	-	-	-	-	-	-	-	-	-	-	-
		Monoterpene hydrocarbons	5.5	15.0	0.6	20.0	16.9	29.9	16.2	8.1	12.9	9.7	4.4	2.2
		Oxygenated monoterpenoids	42.2	72.0	31.1	32.9	65.4	56.9	77.5	63.0	80.6	51.3	35.9	79.4
		Sesquiterpene hydrocarbons	1.1	0.4	1.5	3.0	0.9	0.5	0.2	1.0	0.3	4.0	0.7	1.2
		Oxygenated sesquiterpenoids	39.8	5.7	49.0	23.8	5.3	1.1	0.7	5.2	0.1	8.1	30.4	2.9
		Diterpenoids	0.7	0.0	0.0	0.0	0.0	0.0	0.0	0.0	0.0	0.0	0.0	0.0
		Benzenoid aromatics	0.8	0.2	0.2	0.5	0.1	0.1	0.0	0.7	0.4	0.2	0.2	0.8
		Others	5.1	3.9	6.1	5.6	3.4	6.8	3.1	8.3	4.6	6.0	15.4	2.8
		Total identified	95.2	97.2	88.5	85.8	92.0	95.3	97.7	86.4	99.0	79.2	87.0	89.2

RI_calc_ = Retention index determined with respect to a homologous series of *n*-alkanes on a ZB-5ms column. RI_db_ = Reference retention index from the databases. *A.l.* = *Artemisia ludoviciana*. tr = trace (< 0.05%). - = not detected.

**Table 5:** Enantiomeric distribution (percent) of chiral terpenoid components of the essential oil of *Artemisia ludoviciana*.

Compounds	RI_db_	RI_calc_	*A.l.* B1	*A.l.* B2	*A.l.* B3	*A.l.* C1	*A.l.* C2	*A.l.* T1	*A.l.* T2	*A.l.* T3	*A.l.* U1	*A.l.* U2	*A.l.* U3	*A.l.* U4
(+)-α-Thujene	950	no	-	0.0	-	-	0.0	-	-	0.0	-	-	-	-
(−)-α-Thujene	951	953	-	100.0	-	-	100.0	-	-	100.0	-	-	-	-
(−)-α-Pinene	976	976	-	88.0	-	77.1	59.9	80.7	61.4	81.5	50.6	74.0	83.8	-
(+)-α-Pinene	982	981	-	12.0	-	22.9	40.1	19.3	38.6	18.5	49.4	26.0	16.2	-
(−)-Camphene	998	997	-	99.4	-	100.0	99.3	99.5	90.3	84.2	99.5	100.0	100.0	100.0
(+)-Camphene	1005	1002	-	0.6	-	0.0	0.7	0.5	9.7	15.8	0.5	0.0	0.0	0.0
(+)-Sabinene	1021	1018	-	16.0	-	13.9	79.4	15.4	21.3	14.7	14.9	17.6	35.0	-
(−)-Sabinene	1030	1027	-	84.0	-	86.1	20.6	84.6	78.7	85.3	85.1	82.4	65.0	-
(+)-β-Pinene	1027	1024	-	10.0	2.5	-	-	13.1	18.9	10.2	25.8	-	12.0	-
(−)-β-Pinene	1031	1029	-	90.0	97.5	-	-	86.9	81.1	89.8	74.2	-	88.0	-
(−)-Limonene	1073	1073	-	86.1	-	43.3	91.8	93.2	38.0	-	-	100.0	-	-
(+)-Limonene	1081	1081	-	13.9	-	56.7	8.2	6.8	62.0	-	-	0.0	-	-
(+)-*cis*-Sabinene hydrate	1199	1199	-	9.4	-	-	-	9.4	33.3	12.8	12.1	-	12.5	-
(−)-*cis*-Sabinene hydrate	1202	1202	-	90.6	-	-	-	90.6	66.7	87.2	87.9	-	87.5	-
(−)-Linalool	1228	1227	0.6	-	98.6	19.0	99.0	-	29.2	74.6	73.0	86.0	85.1	95.8
(+)-Linalool	1231	1231	99.4	-	1.4	81.0	1.0	-	70.8	25.4	27.0	14.0	14.9	4.2
(+)-*trans*-Sabinene hydrate	1231	1230	-	43.5	-	-	-	32.3	-	33.0	31.4	-	-	-
(−)-*trans*-Sabinene hydrate	1235	1234	-	56.5	-	-	-	67.7	-	67.0	68.6	-	-	-
(−)-Camphor	1253	1258	100.0	100.0	-	-	100.0	100.0	100.0	38.9	100.0	100.0	100.0	100.0
(+)-Camphor	1259	1261	0.0	0.0	-	-	0.0	0.0	0.0	61.1	0.0	0.0	0.0	0.0
(+)-Terpinen-4-ol	1297	1296	-	34.4	-	0.0	0.0	43.5	58.3	35.2	35.2	-	36.1	45.5
(−)-Terpinen-4-ol	1300	1299	-	65.6	-	100.0	100.0	56.5	41.7	64.8	64.8	-	63.9	54.5
(−)-Lavandulol	1314	1314	100.0	-	100.0	100.0	100.0	-	-	-	-	-	-	-
(+)-Lavandulol	na	no	0.0	-	0.0	0.0	0.0	-	-	-	-	-	-	-
(−)-Borneol	1335	1334	-	100.0	-	-	100.0	100.0	100.0	-	100.0	-	100.0	-
(+)-Borneol	1340	no	-	0.0	-	-	0.0	0.0	0.0	-	0.0	-	0.0	-
(−)-α-Terpineol	1347	1346	82.3	40.9	-	85.2	-	89.1	58.5	86.3	82.0	85.2	83.4	-
(+)-α-Terpineol	1356	1355	17.7	59.1	-	14.8	-	10.9	41.5	13.7	18.0	14.8	16.6	-
(−)-α-Copaene	1381	1382	100.0	-	100.0	100.0	-	-	-	-	-	100.0	-	-
(+)-α-Copaene	na	no	0.0	-	0.0	0.0	-	-	-	-	-	0.0	-	-
(−)-(*E*)-β-Caryophyllene	1461	1463	-	-	100.0	100.0	-	-	-	-	-	100.0	-	100.0
(+)-(*E*)-β-Caryophyllene	na	no	-	-	0.0	0.0	-	-	-	-	-	0.0	-	0.0
(+)-Germacrene D	1519	no	-	0.0	0.0	0.0	0.0	0.0	-	-	-	-	-	0.0
(−)-Germacrene D	1522	1524	-	100.0	100.0	100.0	100.0	100.0	-	-	-	-	-	100.0
(−)-δ-Cadinene	1563	no	0.0	-	0.0	0.0	0.0	-	-	-	-	-	-	0.0
(+)-δ-Cadinene	1576	1578	100.0	-	100.0	100.0	100.0	-	-	-	-	-	-	100.0
(−)-(*E*)-Nerolidol	1677	1677	0.0	87.1	0.0	60.2	100.0	-	38.7	-	-	0.0	0.0	41.8
(+)-(*E*)-Nerolidol	1680	1679	100.0	12.9	100.0	39.8	0.0	-	61.3	-	-	100.0	100.0	58.2

RI_db_ = Retention index from our in-house database. RI_calc_ = Calculated retention index based on a homologous series of *n*-alkanes on a Restek B-Dex 325 capillary column. *A.l. = Artemisia lucoviciana.* na = Reference compound not available. no = not observed. - = compound not detected.

**Table 6:** Chemical composition (percent of total) of the essential oil from the aerial parts of *Gutierrezia sarothrae* from southwestern Idaho.

RI_calc_	RI_db_	Compounds	*G.s.* #1	*G.s.* #2	*G.s.* #3	*G.s.* #4	*G.s.* #5	*G.s.* #6
844	842	Ethyl 2-methylbutyrate	0.3	-	0.2	0.6	0.1	0.3
848	847	Ethyl isovalerate	0.2	-	-	0.1	0.1	0.1
923	924	Ethyl tiglate	-	-	-	0.1	tr	0.1
926	925	α-Thujene	tr	tr	tr	-	tr	tr
933	933	α-Pinene	0.7	1.7	1.0	0.6	0.1	0.8
948	948	α-Fenchene	tr	tr	tr	tr	tr	tr
950	950	Camphene	tr	0.1	0.1	tr	tr	0.1
972	971	Sabinene	0.1	tr	tr	tr	tr	tr
978	978	β-Pinene	6.2	18.4	12.3	5.9	0.5	8.6
987	993	Methyl isoheptanoate	0.1	-	tr	-	tr	0.1
989	989	Myrcene	1.5	0.7	0.7	1.8	1.5	1.1
999	1003	Ethyl hexanoate	0.3	tr	0.2	tr	0.1	0.1
1005	1004	Octanal	-	tr	0.1	-	tr	tr
1007	1006	3-Ethenyl-1,2-dimethylcyclohexa-1,4-diene	-	-	0.2	0.1	0.2	0.1
1008	1007	α-Phellandrene	11.8	7.0	2.2	7.7	3.0	9.2
1009	1009	δ-3-Carene	tr	tr	-	-	tr	tr
1012	1012	Hexyl acetate	-	-	0.2	-	-	-
1017	1017	α-Terpinene	0.2	0.2	0.1	0.1	0.1	0.1
1025	1025	*p*-Cymene	1.8	0.8	0.4	1.2	0.8	1.4
1030	1030	Limonene	11.7	1.4	10.3	11.0	25.4	7.4
1032	1031	β-Phellandrene	0.7	0.8	0.4	0.5	0.2	0.6
1033	1032	1,8-Cineole	1.0	tr	tr	tr	0.6	0.1
1036	1034	(*Z*)-β-Ocimene	20.1	32.1	39.4	28.3	18.8	21.3
1046	1045	(*E*)-β-Ocimene	2.2	3.1	3.2	2.7	1.9	2.2
1058	1057	γ-Terpinene	0.2	0.2	0.1	0.1	0.1	0.1
1070	1069	*cis*-Linalool oxide (furanoid)	-	-	-	-	0.1	-
1085	1086	Terpinolene	0.1	0.3	0.2	0.1	tr	0.2
1086	1086	*trans*-Linalool oxide (furanoid)	-	-	-	-	tr	-
1086	1085	Methyl 6-methylheptanoate	0.1	-	-	-	tr	-
1090	1091	*p*-Cymenene	-	-	-	-	tr	-
1092	1091	Rosefuran	-	tr	0.1	tr	tr	-
1100	1101	Linalool	0.1	tr	tr	0.1	0.2	0.1
1111	1114	Heptyl acetate	-	-	0.1	-	-	-
1120	1119	*endo*-Fenchol	-	tr	tr	tr	-	-
1122	1119	1,3,8-*p*-Menthatriene	0.9	0.6	0.9	0.7	0.6	0.7
1123	1123	Methyl octanoate	-	tr	0.1	-	tr	tr
1125	1124	*cis-p*-Menth-2-en-1-ol	0.2	0.2	0.1	0.2	0.1	0.2
1128	1127	(4*E*,6*Z*)-*allo*-Ocimene	0.8	1.4	1.6	1.2	0.8	0.9
1130	1130	(3*E*,5*E*)-2,6-Dimethyl-1,3,5,7-octatetraene	0.9	0.7	1.0	0.7	0.7	0.7
1143	1142	*trans-p*-Menth-2-en-1-ol	0.1	0.1	0.1	0.1	tr	0.1
1152	1151	Citronellal	tr	tr	0.1	0.1	0.1	0.1
1155	1156	Camphene hydrate	tr	0.1	0.1	tr	-	tr
1163	1164	Pinocarvone	-	tr	tr	-	-	-
1170	1170	Borneol	-	tr	-	tr	-	-
1176	1176	*cis*-Pinocamphone	-	tr	tr	-	-	-
1181	1180	Terpinen-4-ol	0.4	0.4	0.3	0.2	0.2	0.3
1186	1185	Dill ether	0.2	tr	tr	tr	-	0.2
1192	1189	Methyl 6-methyloctanoate	0.1	-	0.1	-	-	tr
1192	1192	Methyl salicylate	-	tr	-	tr	-	-
1194	1196	(4*Z*)-Decenal	-	-	0.2	-	-	-
1196	1195	α-Terpineol	1.0	2.0	1.4	0.8	0.3	1.0
1206	1206	Decanal	-	-	tr	-	-	-
1208	1207	(3*E*)-Octenyl acetate	-	-	0.1	tr	tr	-
1210	1211	Octyl acetate	-	-	0.1	-	-	-
1226	1227	Citronellol	2.2	0.2	0.6	1.7	0.7	1.2
1238	1238	Neral	-	-	tr	-	tr	tr
1267	1268	Geranial	-	-	tr	-	tr	tr
1273	1273	Methyl hydrocinnamate	-	-	-	tr	tr	tr
1306	1307	Methyl (4*Z*)-decenoate	-	-	0.1	-	-	-
1309	1309	4-Vinylguaiacol	-	-	-	-	tr	-
1349	1349	Citronellyl acetate	0.1	tr	0.1	0.1	0.4	0.2
1361	1358	4aα,7α,7aα-Nepetalactone	0.1	tr	tr	0.1	tr	0.1
1373	1374	Methyl *p*-anisate	-	-	tr	-	-	0.1
1375	1375	α-Copaene	0.2	0.1	tr	0.1	tr	0.1
1377	1378	Geranyl acetate	-	-	-	-	tr	-
1384	1384	Methyl (*E*)-cinnamate	0.4	tr	1.9	0.9	3.0	3.2
1387	1387	7-*epi*-Sesquithujene	-	-	-	-	tr	tr
1389	1390	*trans*-β-Elemene	0.1	-	tr	tr	tr	tr
1392	1392	(*Z*)-Jasmone	-	-	tr	-	-	-
1397	1398	4aβ,7α,7aβ-Nepetalactone	tr	tr	tr	tr	tr	tr
1406	1406	α-Gurjunene	tr	-	-	-	-	-
1410	---	Unidentified (RI 1410)	0.6	1.9	0.5	0.1	tr	0.6
1419	1417	(*E*)-β-Caryophyllene	0.2	tr	tr	0.1	0.1	0.1
1429	1433	β-Copaene	tr	-	-	-	-	-
1432	1432	*trans*-α-Bergamotene	-	-	-	-	tr	-
1438	---	2-Tetradecyne	0.1	tr	-	0.1	tr	0.1
1438	1438	Aromadendrene	-	-	tr	-	-	-
1443	---	Unidentified (RI 1443)	5.1	5.7	2.4	0.5	0.3	9.0
1445	1446	cis-Muurola-3,5-diene	tr	tr	-	-	-	-
1448	1450	*trans*-Muurola-3,5-diene	0.1	-	-	tr	tr	0.2
1451	1452	(*E*)-β-Farnesene	-	-	-	-	tr	tr
1455	1454	α-Humulene	0.1	tr	tr	tr	tr	tr
1459	1458	*allo*-Aromadendrene	0.2	tr	0.1	0.1	0.1	0.1
1462	1463	*cis*-Muurola-4(14),5-diene	0.1	tr	tr	tr	tr	tr
1463	---	Unidentified (RI 1463)	0.2	0.3	0.1	8.2	4.0	0.7
1465	1463	γ-Decalactone	0.1	tr	0.2	0.9	0.9	0.2
1467	1469	Ethyl (*E*)-cinnamate	0.1	-	-	0.2	0.1	0.4
1471	1472	*cis*-Cadina-1(6),4-diene	0.4	0.5	0.1	0.2	0.1	0.2
1474	1475	γ-Muurolene	0.6	0.2	0.2	0.3	0.2	0.3
1477	1478	γ-Curcumene	0.1	0.1	tr	0.4	0.3	0.3
1478	1480	Citronellyl isobutyrate	-	-	-	0.1	-	-
1480	1482	*ar*-Curcumene	-	tr	tr	-	-	-
1481	1480	Germacrene D	0.1	0.1	-	0.1	0.1	0.1
1484	1477	*trans*-Cadina-1(6),4-diene	0.1	tr	-	-	-	-
1488	1489	β-Selinene	0.1	tr	tr	0.1	tr	0.1
1491	1490	γ-Amorphene	0.1	tr	0.1	0.1	tr	0.1
1493	1496	Methyl isoeugenol	-	0.2	0.1	-	-	-
1496	1498	*cis*-β-Guaiene	0.2	0.1	0.1	0.1	tr	0.1
1498	1497	α-Muurolene	0.8	0.3	0.2	0.5	0.3	0.5
1509	1510	(*E*)-Lachnophyllum ester	tr	-	0.5	3.1	1.4	0.1
1509	---	Unidentified (RI 1509)	1.6	7.3	0.9	-	-	1.7
1512	1512	γ-Cadinene	-	0.4	0.3	0.7	0.3	0.7
1515	1521	(*Z*)-Lachnophyllum ester	0.7	1.0	0.6	1.5	0.4	1.0
1518	1518	δ-Cadinene	4.1	1.7	1.1	2.8	1.6	2.5
1521	1517	(*E*,*Z*)-Matricaria ester	0.2	1.0	0.7	0.1	7.5	0.7
1521	1519	*trans*-Calamenene	-	tr	-	-	-	-
1522	1521	Zonarene	0.1	0.1	-	0.1	-	0.1
1526	1527	(*Z*,*E*)-Matricaria ester	3.1	0.2	7.4	0.5	9.3	6.4
1527	---	Unidentified (RI 1527)	-	-	-	2.1	0.5	0.2
1531	1533	*trans*-Cadina-1,4-diene	0.2	0.1	-	0.1	0.2	0.1
1536	1538	α-Cadinene	0.2	0.1	0.1	0.2	0.1	0.1
1549	1549	α-Elemol	-	-	-	-	1.4	0.4
1560	1560	(*E*)-Nerolidol	0.2	0.1	-	0.1	0.3	0.2
1576	1574	Germacra-1(10),5-dien-4β-ol	0.2	tr	tr	0.1	0.1	0.1
1576	1576	Spathulenol	-	-	tr	-	-	-
1585	1582	*epi*-Globulol	tr	tr	0.1	0.1	tr	0.1
1594	1594	Viridiflorol	tr	tr	0.1	tr	-	-
1595	1593	Guaiol	-	-	-	-	0.1	-
1614	1614	1,10-di-*epi*-Cubenol	0.2	0.1	0.1	0.1	0.1	0.1
1621	1624	*epi*-γ-Eudesmol	-	-	-	-	0.1	tr
1627	1628	1-*epi*-Cubenol	0.5	0.2	0.1	0.3	0.1	0.3
1632	1632	γ-Eudesmol	-	-	-	-	1.7	0.5
1641	1640	τ-Cadinol	1.8	1.0	0.6	1.3	0.6	1.2
1644	1644	τ-Muurolol	1.8	0.9	0.7	1.4	0.7	1.2
1646	1645	α-Muurolol (=δ-Cadinol)	1.9	0.5	0.2	1.1	0.3	1.0
1655	1655	α-Eudesmol	-	-	-	-	1.8	0.7
1655	1655	α-Cadinol	4.0	2.3	1.6	3.1	3.8	3.1
		Monoterpene hydrocarbons	59.8	69.4	74.1	62.9	54.7	55.6
		Oxygenated monoterpenoids	5.3	3.0	2.8	3.3	2.6	3.4
		Sesquiterpene hydrocarbons	7.8	3.6	2.1	6.0	3.3	5.6
		Oxygenated sesquiterpenoids	10.5	5.0	3.4	7.6	11.0	8.8
		Benzenoid aromatics	0.5	0.2	2.0	1.1	3.0	3.7
		Acetylenes	4.0	2.2	9.2	5.2	18.6	8.3
		Others	1.1	0.0	1.5	1.8	1.1	0.9
		Total identified	89.0	83.4	95.0	88.0	94.4	86.2

RI_calc_ = Retention index determined with respect to a homologous series of *n*-alkanes on a ZB-5ms column. RI_db_ = Reference retention index from the databases. *G.s.* = *Gutierrezia sarothrae*. tr = trace (< 0.05%). - = not detected.

**Table 7:** Enantiomeric distribution of chiral terpenoids in the essential oil from the aerial parts of *Gutierrezia sarothrae*.

Compounds	RI_db_	RI_calc_	*G.s.* #1	*G.s.* #2	*G.s.* #3	*G.s.* #4	*G.s.* #5	*G.s.* #6
(−)-α-Pinene	976	976	86.4	96.2	95.3	89.7	62.4	90.8
(+)-α-Pinene	982	983	13.6	3.8	4.7	10.3	37.6	9.2
(+)-β-Pinene	1027	1027	0.4	0.2	0.2	0.2	2.7	0.3
(−)-β-Pinene	1031	1029	99.6	99.8	99.8	99.8	97.3	99.7
(−)-α-Phellandrene	1050	1051	100.0	100.0	100.0	100.0	100.0	100.0
(+)-α-Phellandrene	1053							
(−)-Limonene	1073	1075	6.5	71.5	9.1	5.2	2.2	8.9
(+)-Limonene	1081	1079	93.5	28.5	90.9	94.8	97.9	91.1
(−)-Linalool	1228	1228	-	-	-	20.6	9.3	-
(+)-Linalool	1231	1232	-	-	-	79.4	90.7	-
(+)-Terpinen-4-ol	1297	1296	31.1	34.4	35.8	32.6	30.4	33.1
(−)-Terpinen-4-ol	1300	1299	68.9	65.6	64.2	67.4	69.6	66.9
(−)-α-Terpineol	1347	1344	83.3	98.1	92.6	81.9	70.8	85.0
(+)-α-Terpineol	1356	1359	16.7	1.9	7.4	18.1	29.2	15.0
(+)-Citronellol	1384	1385	29.8	31.6	32.3	34.0	35.3	31.4
(−)-Citronellol	1384	1386	70.2	68.4	67.7	66.0	64.7	68.6
(+)-δ-Cadinene	1576	1576	100.0	100.0	100.0	100.0	100.0	100.0

RI_db_ = Retention index from our in-house database. RI_calc_ = Calculated retention index based on a homologous series of *n*-alkanes on a Restek B-Dex 325 capillary column. *G.s.* = *Gutierrezia sarothrae*. - = compound not detected.

**Table 8:** Collection and hydrodistillation details for *Ambrosia acanthicarpa*, *Artemisia ludoviciana*, and *Gutierrezia sarothrae* from the Owyhee Mountains of Idaho.

Sample	Collection Site	Collection Date	Mass Aerial Parts (g)	Mass Essential Oil (g)	% Yield
*A.a.* #1	43°8′56″ N, 116°30′58″ W, 1033 m asl	11 August 2023	53.34	2.673	5.011
*A.a.* #2	43°8′56″ N, 116°30′58″ W, 1033 m asl	11 August 2023	60.82	2.651	4.358
*A.a.* #3	43°8′56″ N, 116°30′58″ W, 1033 m asl	11 August 2023	94.85	4.610	4.860
*A.l.* B1	43°7′7″ N, 116°43′51″ W, 1862 m asl	21 July 2023	64.18	1.263	1.968
*A.l.* B2	43°7′7″ N, 116°43′51″ W, 1862 m asl	21 July 2023	76.65	2.534	3.306
*A.l.* B3	43°7′7″ N, 116°43′51″ W, 1862 m asl	21 July 2023	36.82	0.213	0.580
*A.l.* C1	43°7′7″ N, 116°43′51″ W, 1862 m asl	21 July 2023	102.46	2.294	2.239
*A.l.* C2	43°7′7″ N, 116°43′51″ W, 1862 m asl	21 July 2023	109.45	3.086	2.819
*A.l.* T1	43°1′34″ N, 116°43′58″ W, 1033 m asl	11 August 2023	41.74	0.571	1.368
*A.l.* T2	43°1′34″ N, 116°43′58″ W, 1033 m asl	11 August 2023	68.90	1.234	1.791
*A.l.* T3	43°1′34″ N, 116°43′58″ W, 1033 m asl	11 August 2023	92.73	2.297	2.477
*A.l.* U1	43°1′34″ N, 116°43′58″ W, 1033 m asl	11 August 2023	102.02	2.741	2.687
*A.l.* U2	43°1′34″ N, 116°43′58″ W, 1033 m asl	11 August 2023	67.98	1.223	1.798
*A.l.* U3	43°1′34″ N, 116°43′58″ W, 1033 m asl	11 August 2023	83.90	2.112	2.517
*A.l.* U4	43°1′34″ N, 116°43′58″ W, 1033 m asl	11 August 2023	85.52	2.158	2.524
*G.s.* #1	43°8′56″ N, 116°30′58″ W, 1033 m asl	11 August 2023	100.64	4.058	4.032
*G.s.* #2	43°8′56″ N, 116°30′58″ W, 1033 m asl	11 August 2023	88.74	3.638	4.100
*G.s.* #3	43°8′56″ N, 116°30′58″ W, 1033 m asl	11 August 2023	91.72	3.376	3.681
*G.s.* #4	43°8′56″ N, 116°30′58″ W, 1033 m asl	11 August 2023	107.85	4.386	4.066
*G.s.* #5	43°8′56″ N, 116°30′58″ W, 1033 m asl	11 August 2023	81.59	3.758	4.606
*G.s.* #6	43°8′56″ N, 116°30′58″ W, 1033 m asl	11 August 2023	98.60	4.080	4.138

## Data Availability

All data are available in the manuscript and Supplementary Materials.

## Supplementary Materials

The following supporting information can be downloaded at: https://www.mdpi.com/article/10.3390/molecules29061383/s1, Figure S1: Mass spectra of major unidentified components in the essential oils of *Artemisia ludoviciana*; Figure S2: Mass spectra of major unidentified components in the essential oils of *Gutierrezia sarothrae*. Table S1: Instrument details for the gas chromatographic analyses of *Ambrosia acanthicarpa*, *Artemisia ludoviciana*, and *Gutierrezia sarothrae*.
