# Supplementary material for: The Essential Oil Compositions of Ambrosia acanthicarpa Hook., Artemisia ludoviciana Nutt., and Gutierrezia sarothrae (Pursh) Britton & Rusby (Asteraceae) from the Owyhee Mountains of Idaho
Source: Molecules. 2024 Mar 20;29(6):1383. doi: 10.3390/molecules29061383 (PMC10976104; doi:10.3390/molecules29061383)
Supplement: Supplementary file 1 [file molecules-29-01383-s001.zip › Supplementary Figure S1.pdf]

Supplementary Figure S1. Mass spectra of unidentified components in the essential oils of *Artemisia ludoviciana*.

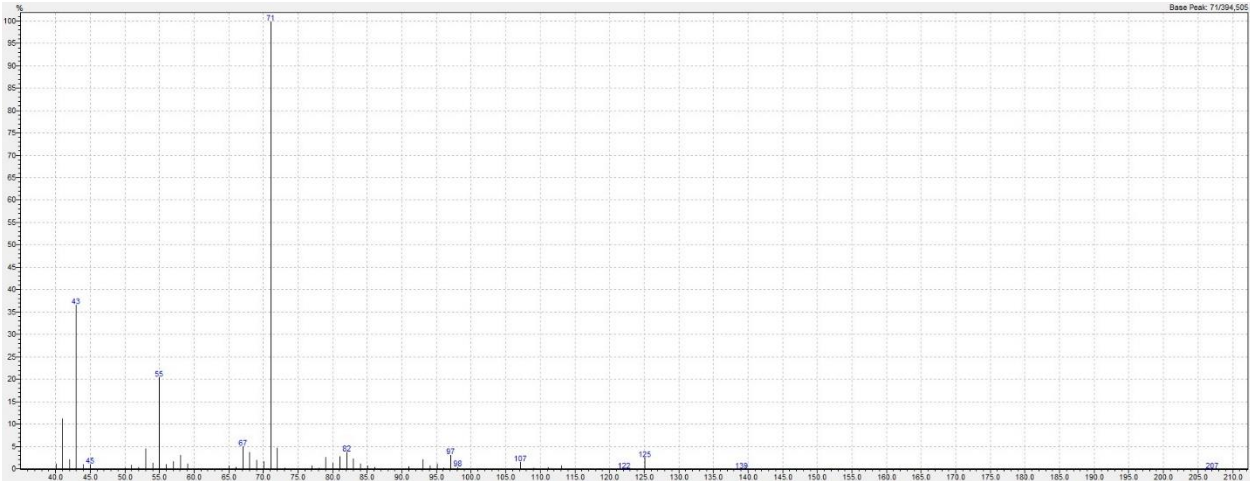

Artemisia ludoviciana Unidentified (RI 1000). MS(EI): 125(3%), 107(2%), 97(3%), 82(4%), 71(100%), 55(20%), 43(37%), 41(11%).

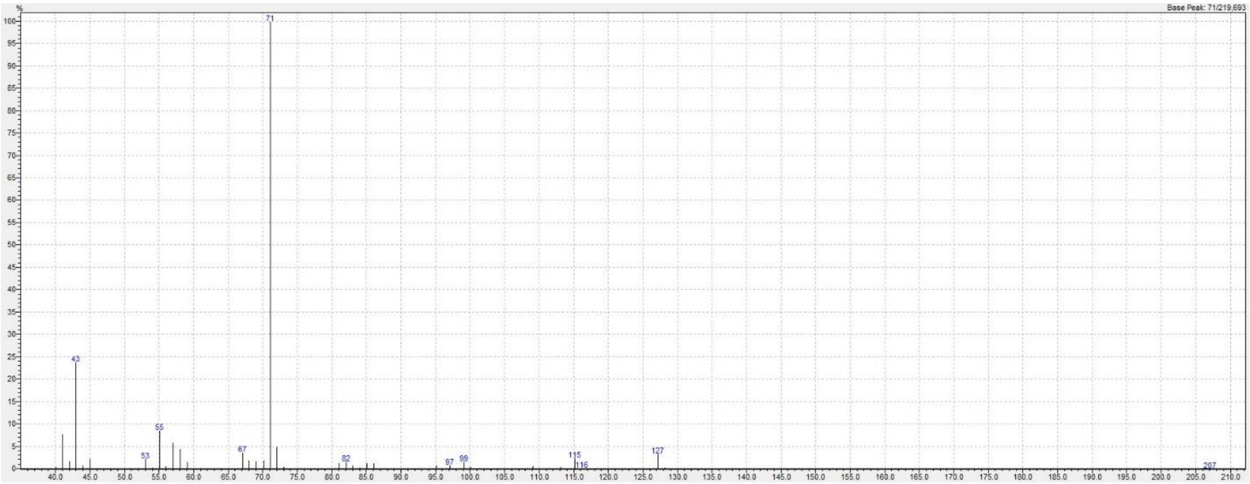

Artemisia ludoviciana Unidentified (RI 1009). MS(EI): 127(4%), 115(3%), 99(2%), 71(100%), 55(8%), 43(24%), 41(8%).

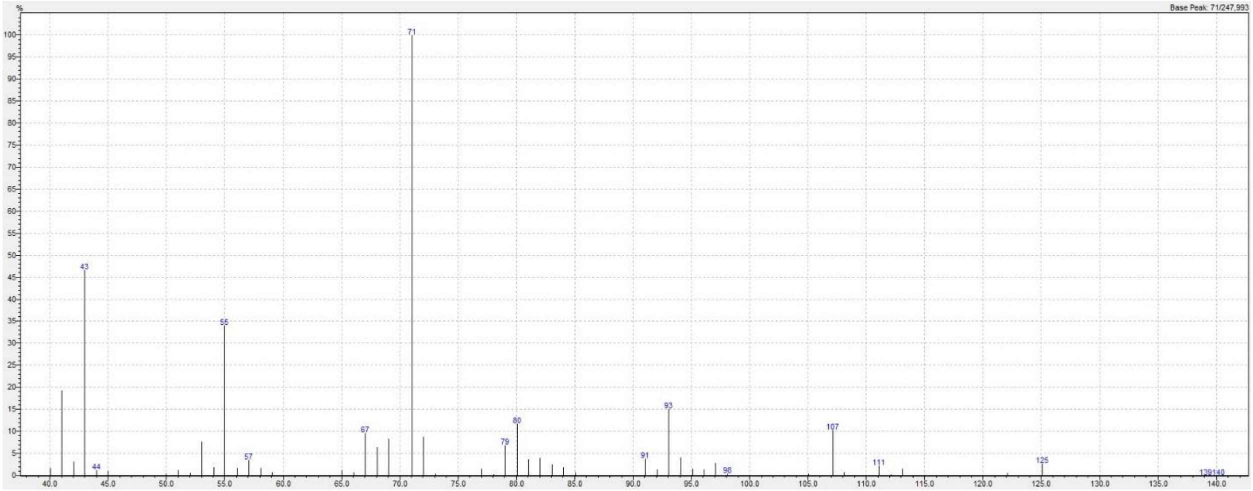

Artemisia ludoviciana Unidentified (RI 1016). MS(EI): 125(3%), 107(10%), 93(15%), 80(12%), 71(100%), 55(34%), 43(47%), 41(19%).

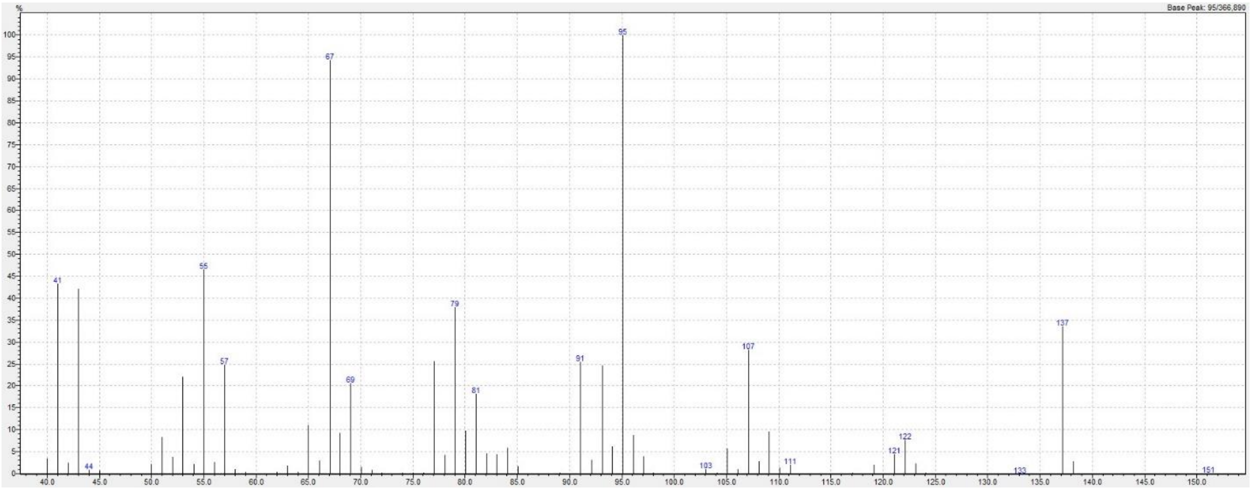

Artemisia ludoviciana Unidentified (RI 1049). MS(EI): 137(34%), 122(8%), 107(29%), 95(100%), 93(25%), 91(26%), 79(38%), 67(94%), 57(25%), 55(47%), 43(42%), 41(44%).

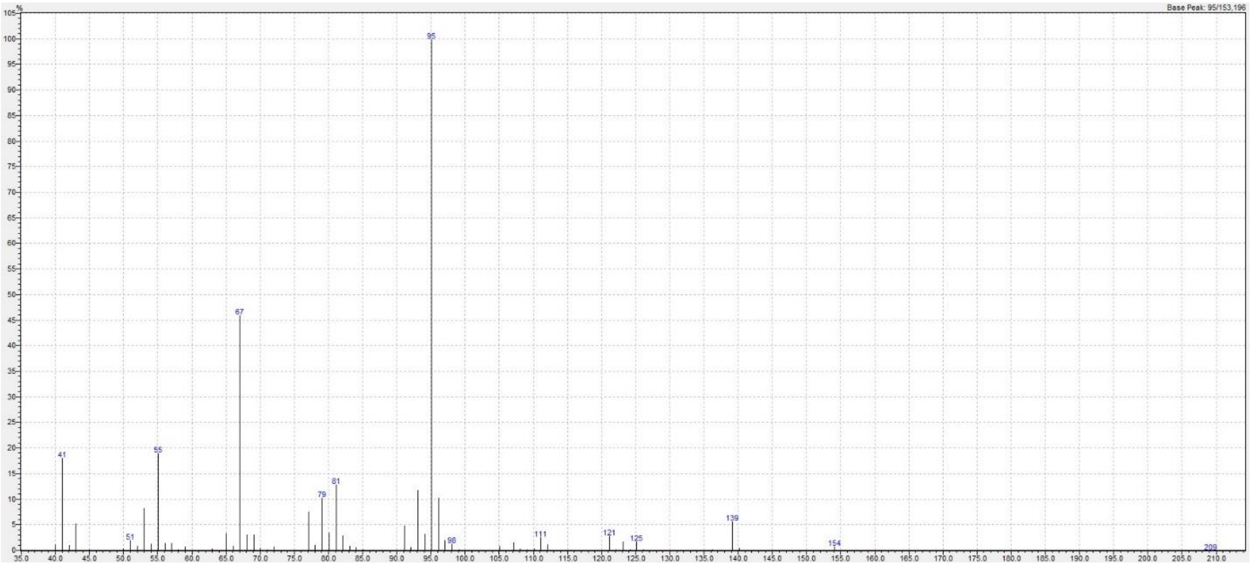

Artemisia ludoviciana Unidentified (RI 1132). MS(EI): 154(2%), 139(6%), 95(100%), 81(13%), 67(46%), 55(20%), 41(18%).

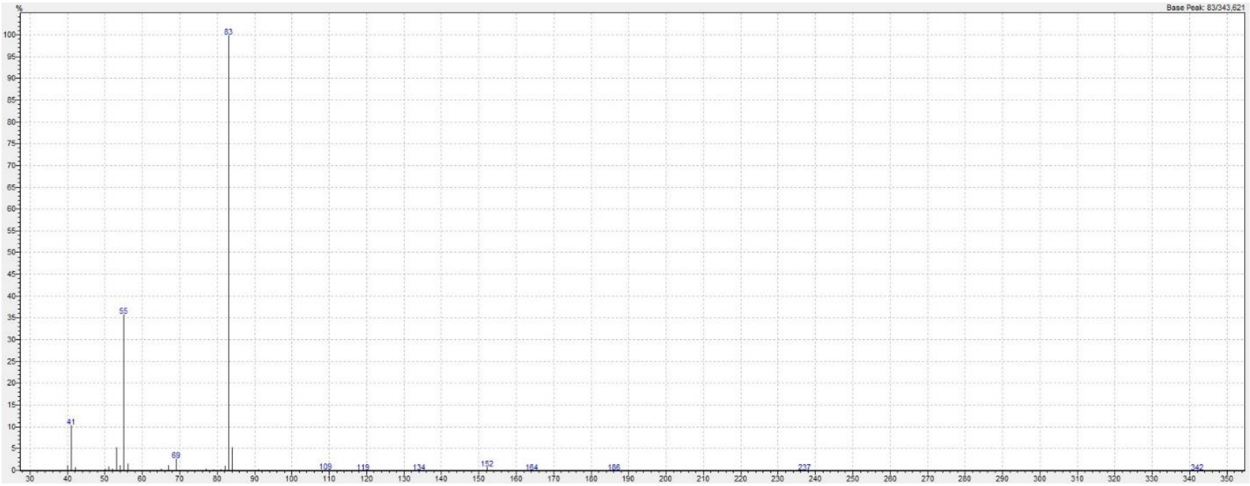

Artemisia ludoviciana Unidentified (RI 1186). MS(EI): 152(1%), 83(100%), 69(3%), 55(36%), 41(10%).

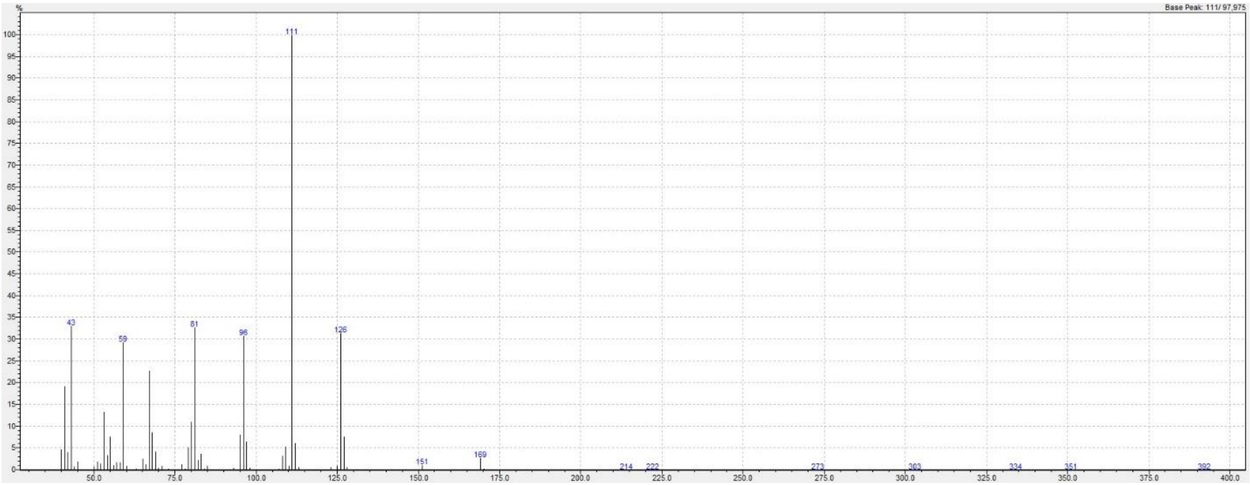

Artemisia ludoviciana Unidentified (RI 1211). MS(EI): 169(3%), 126(32%), 111(100%), 96(31%), 81(33%), 67(23%), 59(29%), 43(33%).

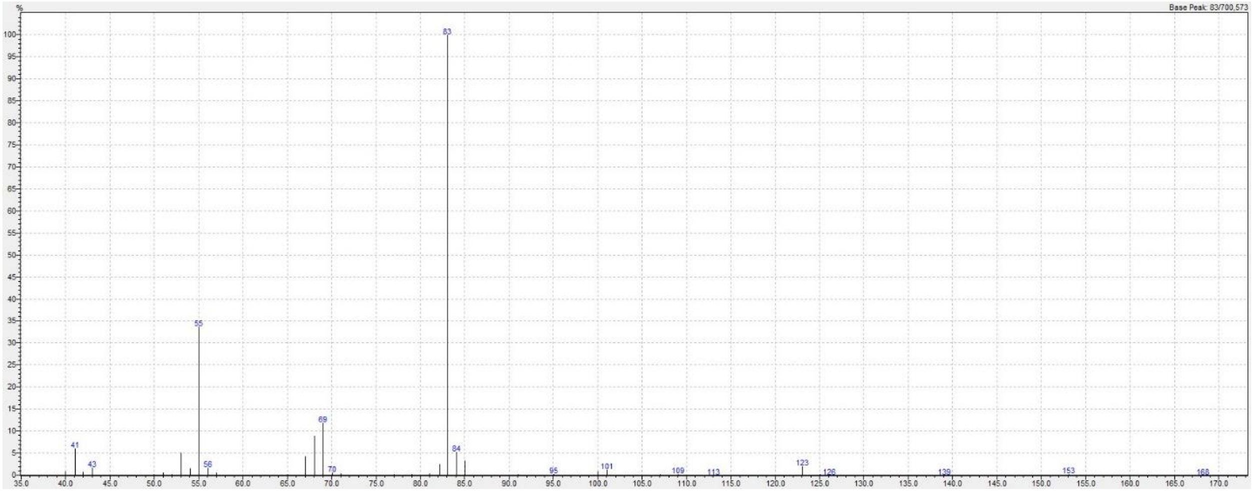

Artemisia ludoviciana Unidentified (RI 1236). MS(EI): 123(2%), 83(100%), 69(12%), 55(34%), 41(6%).

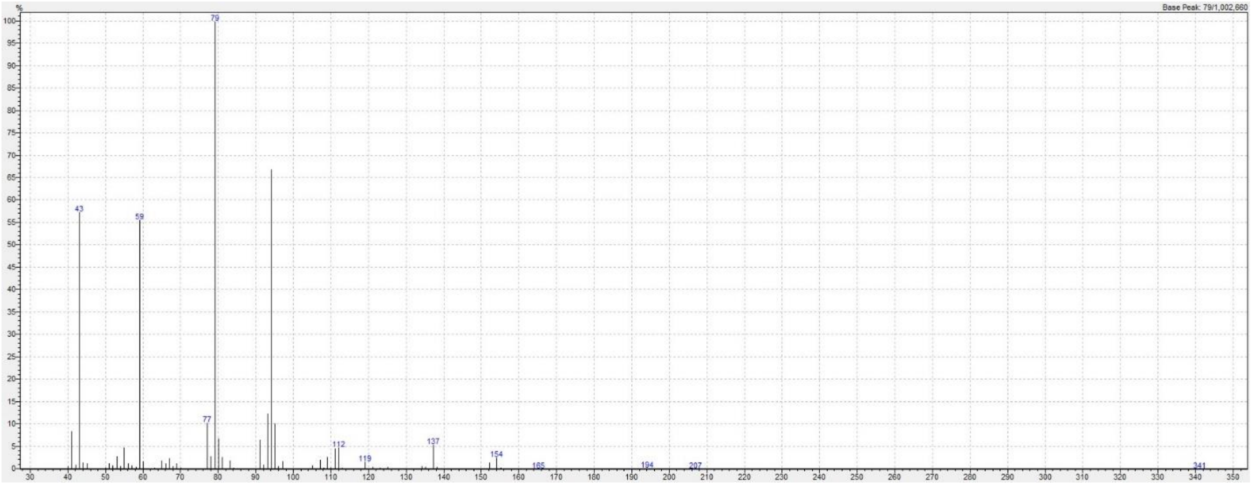

Artemisia ludoviciana Unidentified (RI 1416). MS(EI): 154(3%), 137(5%), 112(5%), 94(66%), 79(100%), 59(55%), 43(57%).

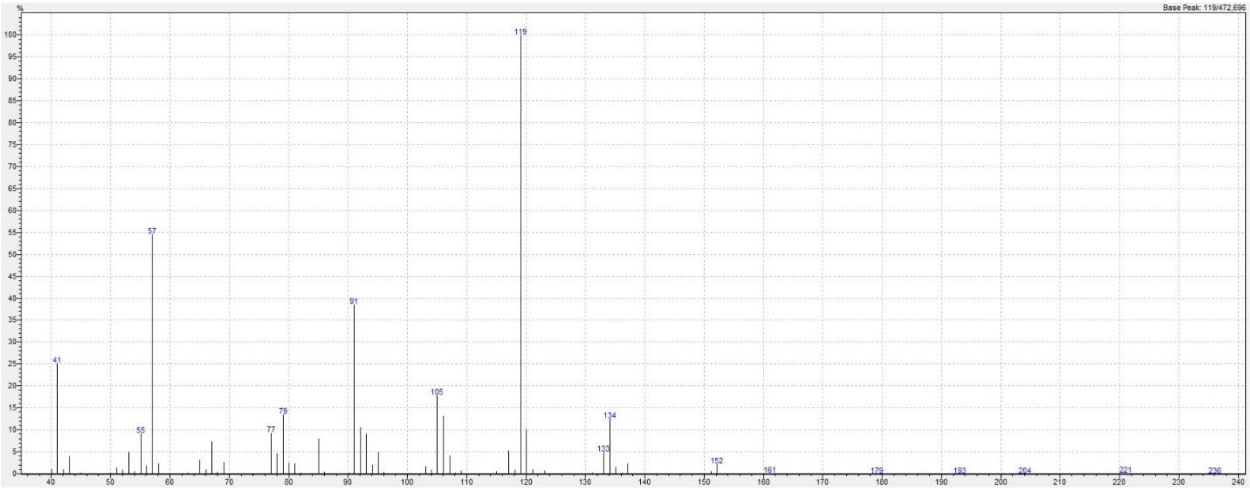

Artemisia ludoviciana Unidentified (RI 1462). MS(EI): 152(3%), 134(13%), 119(100%), 105(18%), 91(38%), 79(13%), 57(55%), 41(25%).

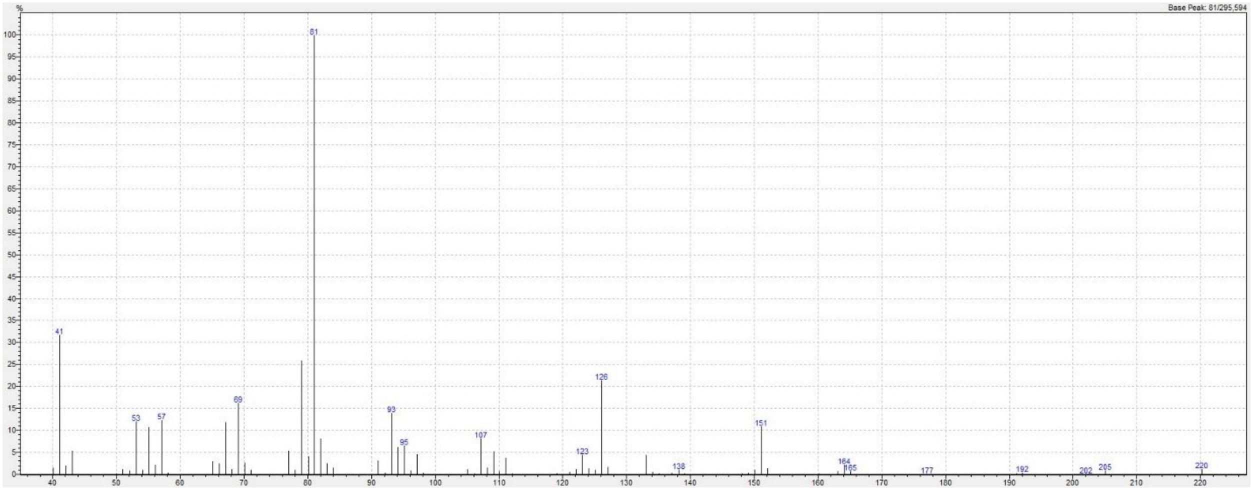

Artemisia ludoviciana Unidentified (RI 1598). MS(EI): 220(2%), 164(3%), 151(11%), 126(22%), 107(8%), 93(14%), 81(100%), 79(26%), 69(16%), 57(13%), 55(11%), 53(12%), 41(32%).

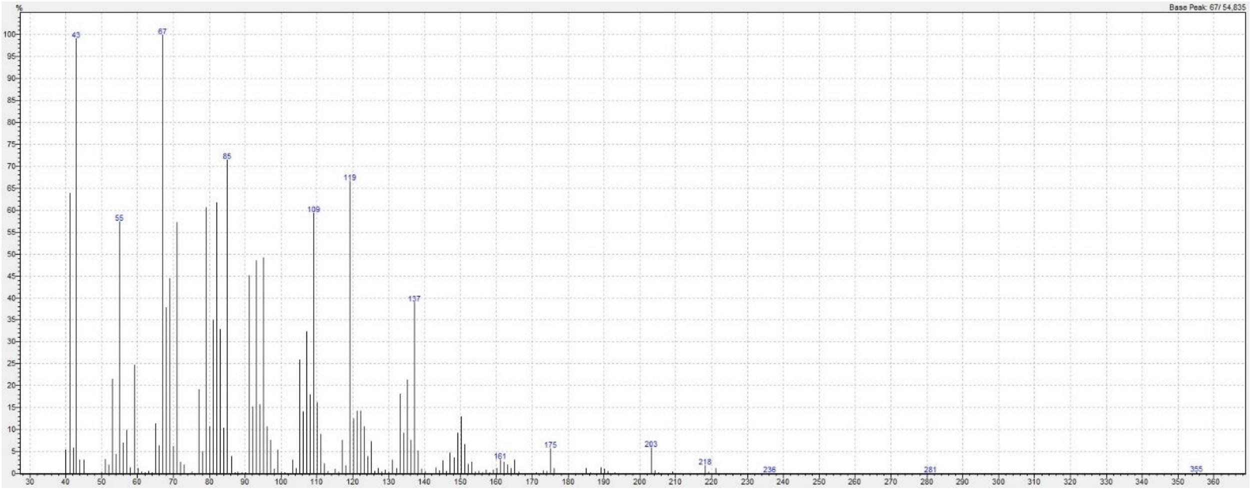

Artemisia ludoviciana Unidentified (RI 1619). MS(EI): 218(2%), 203(6%), 175(6%), 137(40%), 119(67%), 109(60%), 85(72%), 82(62%), 79(61%), 71(57%), 67(100%), 55(58%), 43(99%), 41(64%).

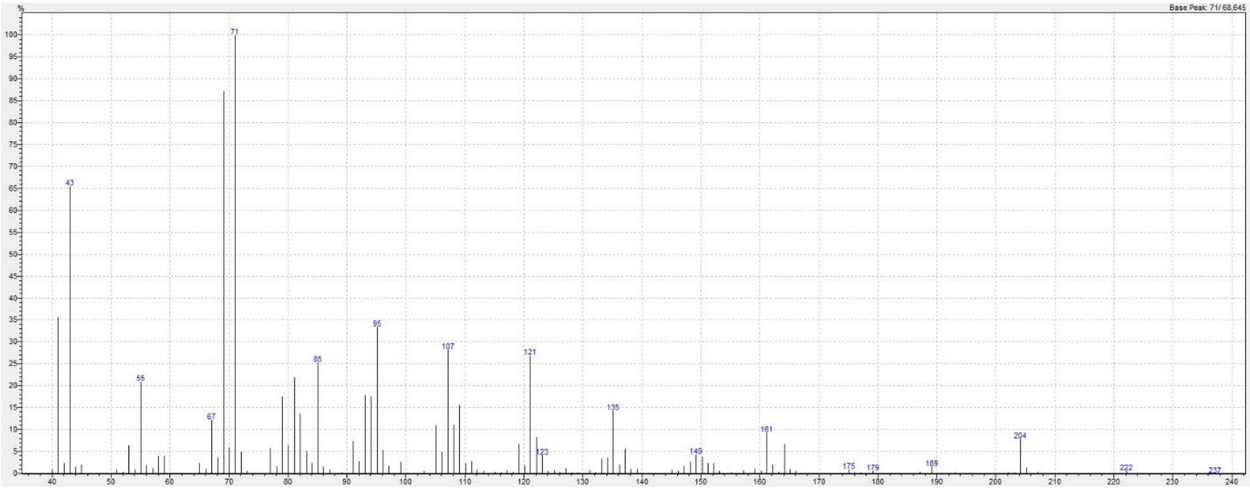

Artemisia ludoviciana Unidentified (RI 1654). MS(EI): 222(1%), 204(8%), 161(10%), 135(15%), 121(27%), 107(29%), 95(33%), 85(25%), 71(100%), 69(87%), 55(21%), 43(65%), 41(35%).

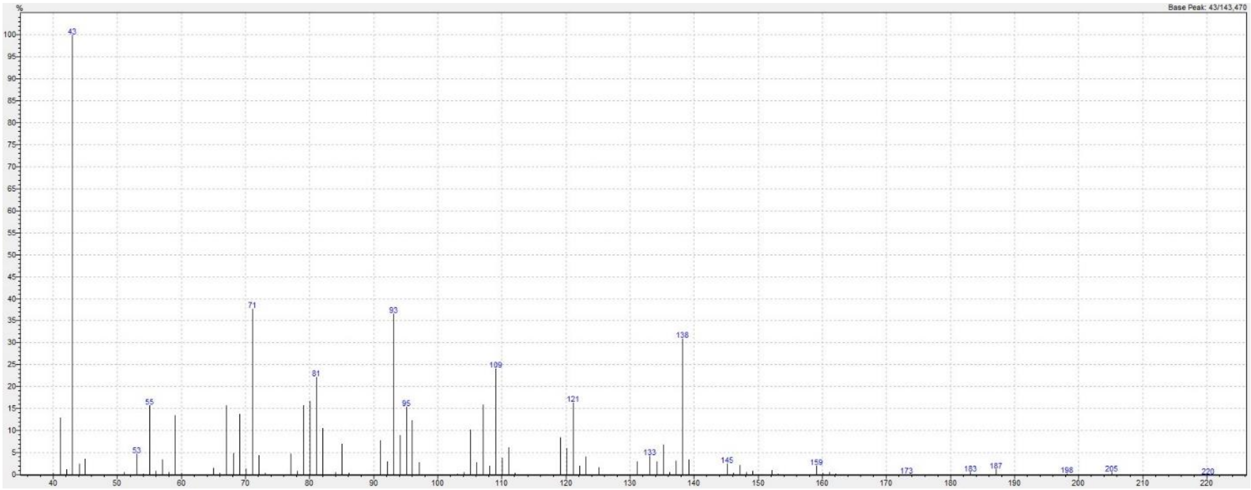

Artemisia ludoviciana Unidentified (RI 1671). MS(EI): 220(1%), 205(1%), 138(31%), 121(16%), 109(25%), 93(37%), 81(22%), 71(38%), 59(13%), 55(15%), 43(100%), 41(13%).

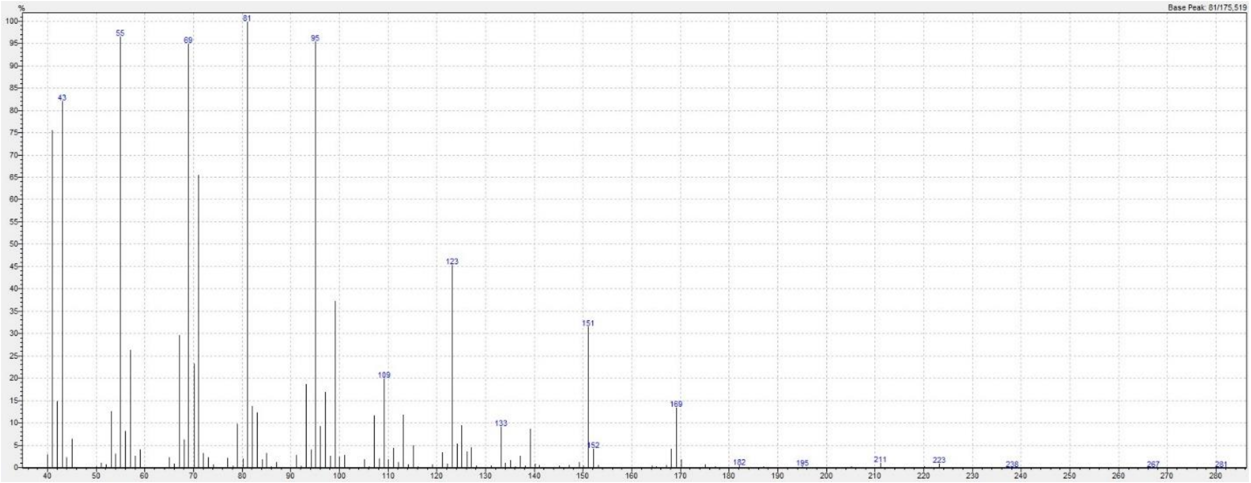

Artemisia ludoviciana Unidentified (RI 1687). MS(EI): 169(14%), 151(32%), 123(45%), 99(37%), 95(95%), 81(100%), 71(65%), 69(95%), 55(97%), 43(83%), 41(75%).

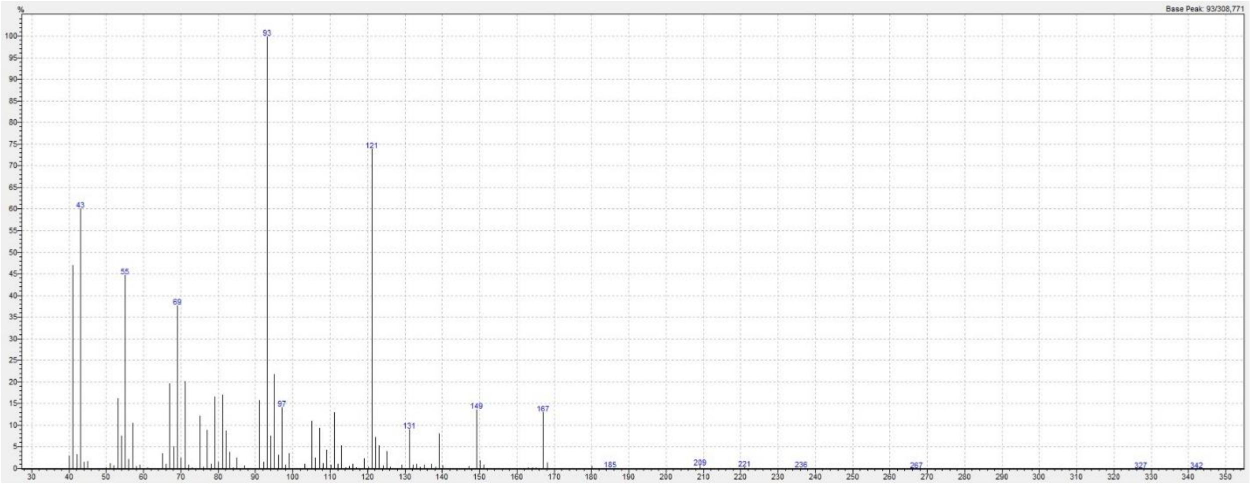

Artemisia ludoviciana Unidentified (RI 1780). MS(EI): 167(13%), 149(14%), 121(74%), 93(100%), 69(38%), 55(45%), 43(60%), 41(47%).
