# Supplementary material for: The Essential Oil Compositions of Ambrosia acanthicarpa Hook., Artemisia ludoviciana Nutt., and Gutierrezia sarothrae (Pursh) Britton & Rusby (Asteraceae) from the Owyhee Mountains of Idaho
Source: Molecules. 2024 Mar 20;29(6):1383. doi: 10.3390/molecules29061383 (PMC10976104; doi:10.3390/molecules29061383)
Supplement: Supplementary file 1 [file molecules-29-01383-s001.zip › Supplementary Figure S2.pdf]

Supplementary Figure S2. Mass spectra of unidentified components in the essential oils of *Gutierrezia sarothrae*.

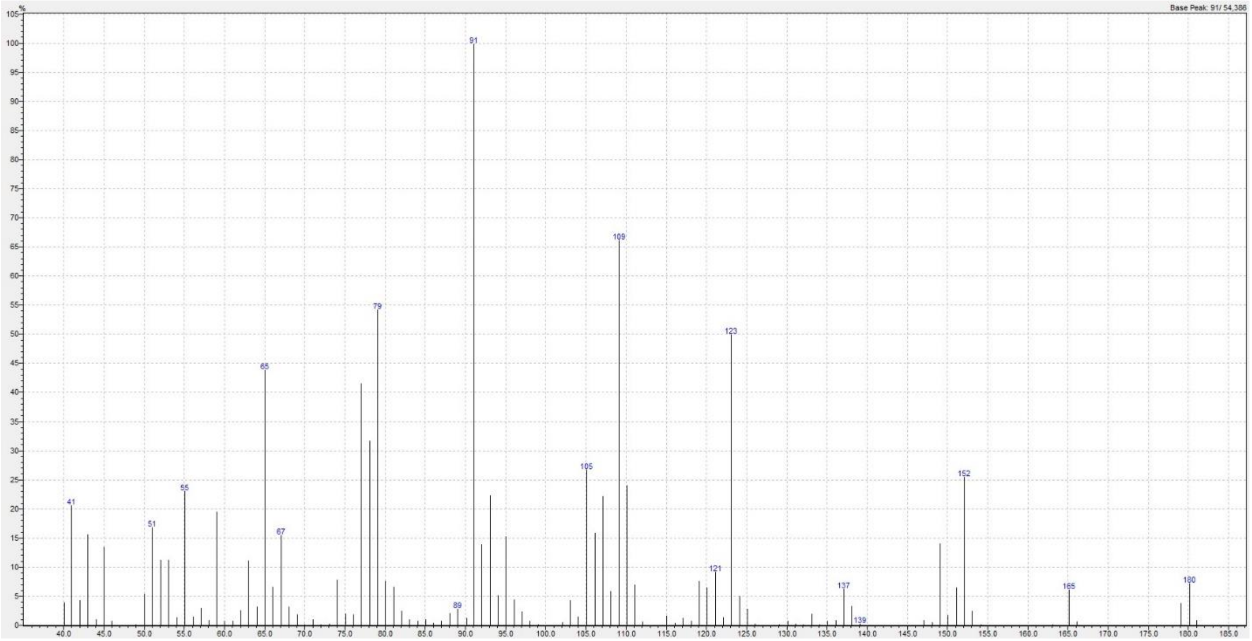

Gutierrezia sarothrae Unidentified (RI 1410). MS(EI): 180(8%), 165(7%), 152(26%), 123(50%), 109(67%), 91(100%), 79(54%), 65(44%), 55(23%), 41(21%).

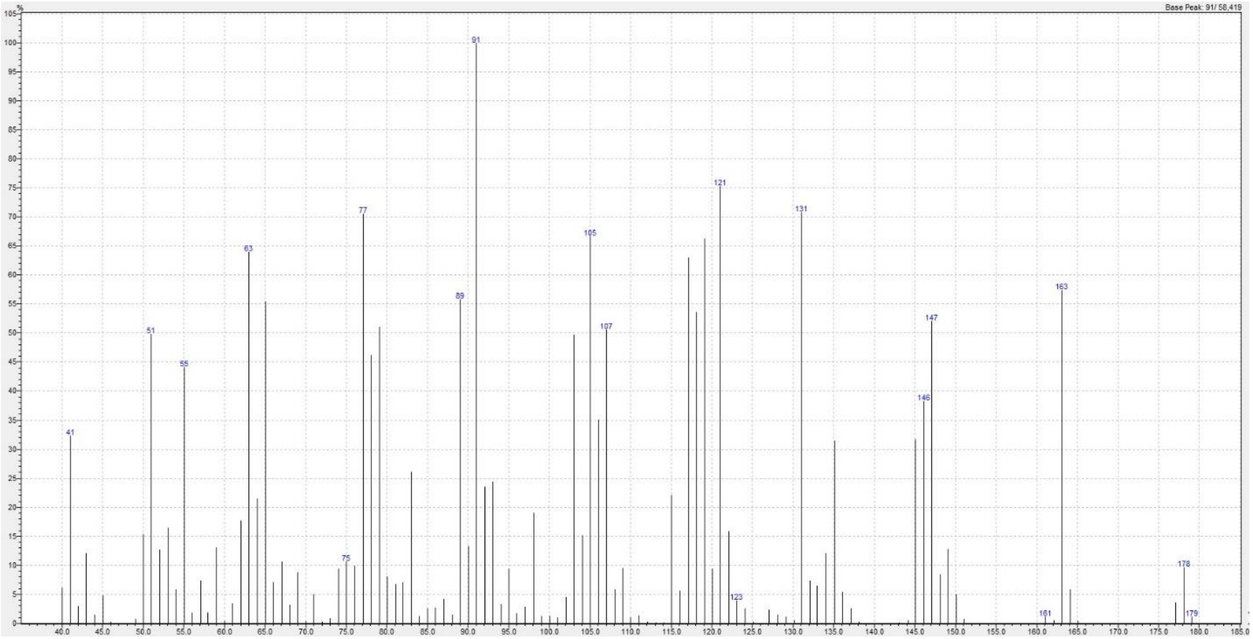

Gutierrezia sarothrae Unidentified (RI 1443). MS(EI): 178(10%), 163(58%), 147(53%), 131(71%), 121(75%), 105(66%), 91(100%), 77(71%), 63(64%), 55(44%), 51(50%), 41(33%).

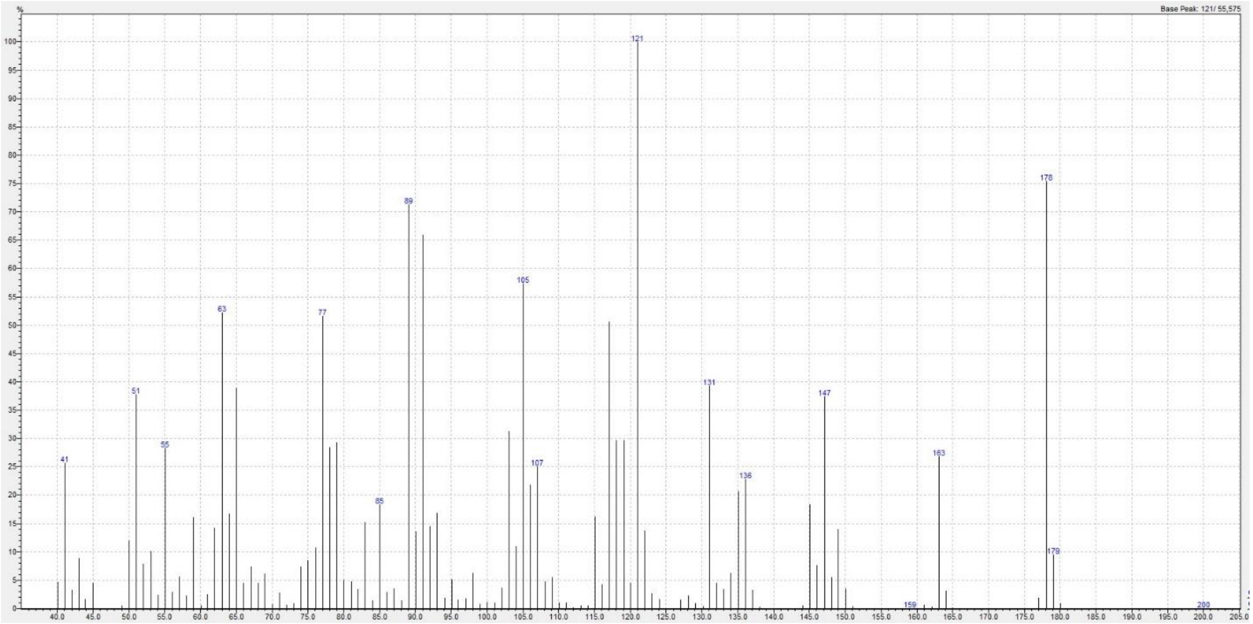

Gutierrezia sarothrae Unidentified (RI 1463). MS(EI): 178(70%), 163(27%), 147(38%), 131(39%), 121(100%), 105(58%), 91(66%), 89(72%), 77(52%), 63(53%), 55(28%), 51(38%), 41(26%).

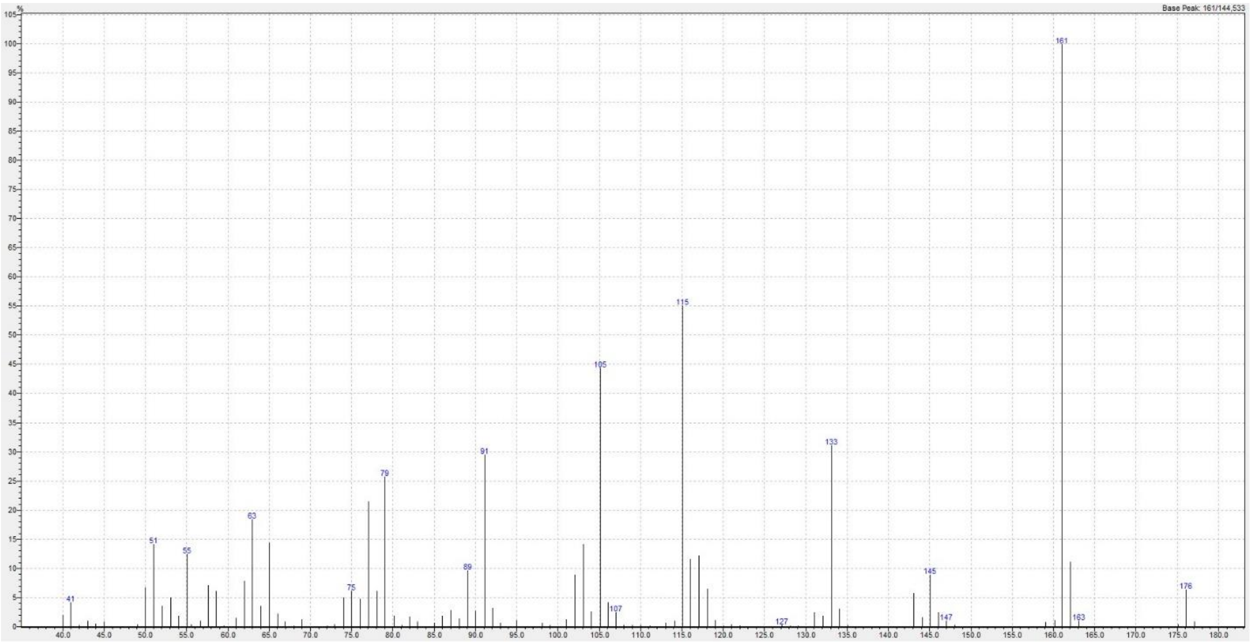

Gutierrezia sarothrae Unidentified (RI 1509). MS(EI): 176(7%), 161(100%), 145(9%), 133(32%), 115(55%), 105(45%), 91(29%), 79(26%), 63(18%), 55(13%), 51(14%), 41(4%).

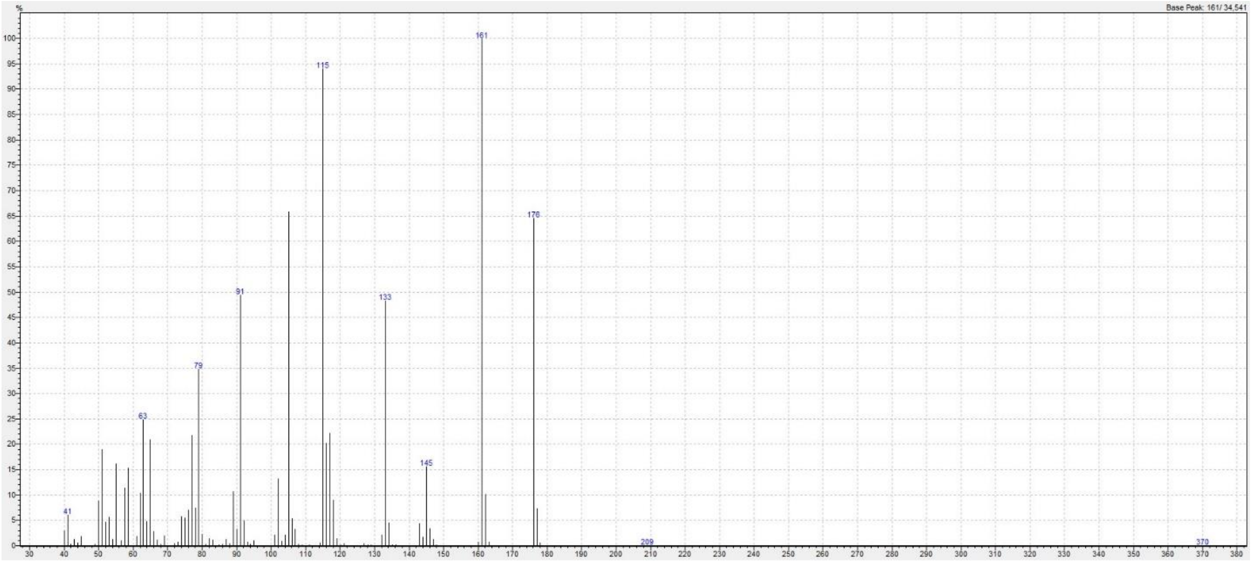

Gutierrezia sarothrae Unidentified (RI 1527). MS(EI): 176(65%), 161(100%), 145(16%), 133(48%), 115(94%), 105(66%), 91(49%), 79(35%), 63(25%), 51(18%), 41(6%).
