# Supplementary material for: The Essential Oil Compositions of Ambrosia acanthicarpa Hook., Artemisia ludoviciana Nutt., and Gutierrezia sarothrae (Pursh) Britton & Rusby (Asteraceae) from the Owyhee Mountains of Idaho
Source: Molecules. 2024 Mar 20;29(6):1383. doi: 10.3390/molecules29061383 (PMC10976104; doi:10.3390/molecules29061383)
Supplement: Supplementary file 1 [file molecules-29-01383-s001.zip › Supplementary Table S1.pdf]

Supplementary Table S1. Instrument details for the gas chromatographic analyses of *Ambrosia acanthicarpa*, *Artemisia ludoviciana*, and *Gutierrezia sarothrae*.

**Gas Chromatography - Mass Spectrometry (GC-MS)**	
Instrument	Shimadzu GC-MS-QP2010 Ultra (Shimadzu Scientific Instruments, Columbia, MD, USA)
GC Column	Zebron ZB-5ms fused silica capillary column (60 m × 0.25 mm × 0.25 μm film thickness) (Phenomenex, Torrance, CA, USA)
MS Detector Conditions	Electron impact (EI) mode, electron energy = 70 eV, a scan = 40–400 atomic mass units, scan rate = 3.0 scans/second
Carrier Gas, Conditions	Helium, column head pressure = 208.3 kPa, flow rate = 2.00 mL/min
Injector, Detector Temperatures	Injector temperature = 260 °C, interface temperature = 260 °C, ion source temperature = 260 °C
GC Oven Temperature Program	Initial temperature = 50 °C, ramp 2 °C/min to 260 °C, hold 260 °C for 5 min
Sample Concentration, Volume Injected	5% (in dichloromethane), 0.1 μL volume
Split Mode	24.5 : 1.0
**Gas Chromatography - Flame Ionization Detection (GC-FID)**	
Instrument	Shimadzu GC 2010 with FID (Shimadzu Scientific Instruments, Columbia, MD, USA)
GC Column	Zebron ZB-5 GC column (60 m × 0.25 mm × 0.25 μm film thickness) (Phenomenex, Torrance, CA,
Carrier Gas, Conditions	Helium, column head pressure = 208.3 kPa, flow rate = 2.00 mL/min
Injector, Detector Temperatures	260 °C
GC Oven Temperature Program	Initial temperature = 50 °C, ramp 2 °C/min to 260 °C, hold 260 °C for 5 min
Sample Concentration, Volume Injected	5% (in dichloromethane), 0.1 μL volume
Split Mode	24.5 : 1.0
**Chiral Gas Chromatography - Mass Spectrometry**	
Instrument	Shimadzu GCMS-QP2010S (Shimadzu Scientific Instruments, Columbia, MD, USA)

GC Column	Restek B-Dex 325 chiral GC column (30 m × 0.25 mm × 0.25 μm film thickness) ((Restek Corp., Bellefonte, PA, USA)
MS Detector Conditions	Electron impact (EI) mode, electron energy = 70 eV, a scan = 40–400 atomic mass units, scan rate = 3.0 scans/second
Carrier Gas, Conditions	Helium, column head pressure = 53.6 kPa, flow rate = 1.00 mL/min
Injector, Detector Temperatures	Injector temperature = 240 °C, interface temperature = 240 °C, ion source temperature = 240 °C
GC Oven Temperature Program	Initial temperature = 50 °C, hold for 5 min, ramp 1 °C/min to 100 °C, ramp 2 °C/min to 220 °C
Sample Concentration, Volume Injected	5% (in dichloromethane), 0.3 μL volume
Split Mode	24.0 : 1.0
